# Definition of a systematic review used in overviews of systematic reviews, meta-epidemiological studies and textbooks

**DOI:** 10.1186/s12874-019-0855-0

**Published:** 2019-11-04

**Authors:** Marina Krnic Martinic, Dawid Pieper, Angelina Glatt, Livia Puljak

**Affiliations:** 1 Department of Otorhinolaryngology, University Hospital Split, Split, Croatia; 2 Institute for Research in Operative Medicine (IFOM), Witten/Herdecke University, Cologne, Germany; 3 Center for Evidence-Based Medicine and Health Care, Catholic University of Croatia, Ilica 242, 10000 Zagreb, Croatia

**Keywords:** Systematic review, Definition, Research methodology

## Abstract

**Background:**

A standard or consensus definition of a systematic review does not exist. Therefore, if there is no definition about a systematic review in secondary studies that analyse them or the definition is too broad, inappropriate studies might be included in such evidence synthesis. The aim of this study was to analyse the definition of a systematic review (SR) in health care literature, elements of the definitions that are used and to propose a starting point for an explicit and non-ambiguous SR definition.

**Methods:**

We included overviews of systematic reviews (OSRs), meta-epidemiological studies and epidemiology textbooks. We extracted the definitions of SRs, as well as the inclusion and exclusion criteria that could indicate which definition of a SR the authors used. We extracted individual elements of SR definitions, categorised and quantified them.

**Results:**

Among the 535 analysed sources of information, 188 (35%) provided a definition of a SR. The most commonly used reference points for the definitions of SRs were Cochrane and the PRISMA statement. We found 188 different elements of SR definitions and divided them into 14 categories. The highest number of SR definition elements was found in categories related to searching (*N* = 51), analysis/synthesis (*N* = 23), overall methods (*N* = 22), quality/bias/appraisal/validity (*N* = 22) and aim/question (*N* = 13). The same five categories were also the most commonly used combination of categories in the SR definitions.

**Conclusion:**

Currently used definitions of SRs are vague and ambiguous, often using terms such as clear, explicit and systematic, without further elaboration. In this manuscript we propose a more specific definition of a systematic review, with the ultimate aim of motivating the research community to establish a clear and unambiguous definition of this type of research.

## Background

In 1990, the term evidence-based medicine (EBM) was coined [1]. It was hailed as a new approach for teaching and practising clinical medicine [2], incorporating “the best available external clinical evidence from a systematic search” [3]. When it comes to the best available evidence about treatment, randomised controlled trials (RCTs) and a systematic review (SR)/meta-analysis are considered the “gold standard” [1].

The EBM movement has been widely adopted, and evidence syntheses are regularly used to support clinical guidelines and recommendations for practice. However, it has been suggested that the EBM might be a movement in crisis [4], as there is “too much evidence” [4]. A study published in 2016 indicated that more than 8000 systematic reviews were being indexed annually in MEDLINE, corresponding to a three-fold increase over the last decade [5]. A search conducted in October 2019 showed that more than 15,000 studies published in 2018 were marked with a systematic review tag in PubMed.

Furthermore, some SRs might actually be misleading, redundant and conflicted [6]. A recent overview of systematic reviews found 12 systematic reviews and two major guidelines about thrombolytic therapy for pulmonary embolism published within less than 2 years. The results of those evidence syntheses were discordant, and the benefit-to-risk ratio was elusive [7]. Inclusion and exclusion criteria played a part in the origin of the discordant results [7].

Just as different inclusion and exclusion criteria might be a problem when conducting a systematic review, the same can happen in overviews of systematic reviews (OSRs) or other types of studies analysing systematic reviews, where results will depend upon inclusion criteria. The problem here is that a standard or consensus definition of a systematic review does not exist.

For example, in a study that reported about the increasing popularity of SRs, Page et al. [5] used the PRISMA-P explanation of a SR [8]. Using a definition when searching for SRs is important because there are studies that may call themselves SRs but are not SRs; we can only speculate that authors use a descriptor SR to label their studies because they are not aware of what a systematic review is, or because systematic reviews are considered to be a higher standard of review.

Therefore, if there is no definition about a systematic review in secondary studies that analyse them or the definition is too broad, inappropriate studies might be included in such evidence synthesis. The aim of this study was to explore and analyse the definition of a systematic review (SR) in health care literature, the elements of definitions that are used and propose a starting point for a new, explicit SR definition.

## Methods

This was a methodological study, for which we developed a protocol a priori. The study protocol is available from the corresponding author on request.

### Included studies

We aimed to collect definitions of systematic reviews in the health care literature. As numerous collections of SRs have already been published in the past, we relied on existing resources. We used three different sources: i) OSRs about healthcare interventions, ii) studies that have analysed the methodological quality of systematic reviews and iii) relevant textbooks/internet sources that define systematic reviews.

### Search

We included OSRs and methodological studies identified previously by Pieper et al. [9, 10]. We used a validated filter for retrieval [11]. Additionally, we searched for EBM-related and epidemiology handbooks published in English or German. There was no systematic search for the handbooks. We compiled a list of relevant handbooks known to us, using the same methodological approach as described by other authors in similar projects [12].

Furthermore, we searched Google Scholar between the 24th January, 2018 and the 7th February, 2018 using the following search phrases: “definition of a systematic review”, “definition of systematic review”, “definition of the systematic review”, “defined a systematic review”, “defined the systematic review”, “systematic review was defined”. Those phrases were used to search any part of the manuscript – without any restrictions. We analysed the first 50 search results for each search phrase if there were more than 50 search phrases retrieved for a phrase. We excluded duplicate manuscripts found by searching multiple sources before starting the analysis.

### Data extraction analysis

We piloted a data extraction form in Microsoft Excel on a sample of ten manuscripts. Two authors piloted the data extraction form (LP, MKM). Furthermore, based on the advice of a third author (DP), the form was further refined. Thus, in an iterative process between the authors, the form was modified where necessary to avoid any misunderstandings or later disagreements.

We extracted the following information: i) whether the analysed literature sources reported a definition of a systematic review and ii) inclusion and exclusion criteria defining systematic reviews. We extracted the relevant exclusion criteria when they had explicit statements about studies that were not included because certain aspects of them were not considered to be characteristics of a systematic review.

When we found a definition or inclusion/exclusion criteria defining systematic reviews, the text was extracted verbatim. Subsequently, from those definitions and the inclusion/exclusion criteria, we extracted elements of a systematic review definition. The definition elements were defined as distinct methodological components and their attributes. Elements described with similar adjectives were not combined; instead, we presented all unique elements separately in order to present a wide variety of adjectives and attributes used in the definitions of SRs. We did not use an a priori defined list of those elements; instead, we presented elements that we found in the analysed sources of information and we kept expanding the list of elements as we found new variations of the elements of the SR definition.

One of the elements of a definition we used was the presence of a meta-analysis (MA), but only if the authors explicitly indicated that the MA was considered as defining characteristic of a SR. For example, in a study published in 2013, Aziz wrote explicitly that SRs without MA were not included because “these were not considered SRs” [13].

Extracted individual elements of the SR definition were then categorised into groups. For example, if a SR definition was: “systematic search”, “reproducible search” or “keywords searched”, those elements were sorted into a category called “Search”. The process of forming categorisations was iterative between the authors until we reached a consensus about the categories that will be used.

We extracted reference(s) for a definition of a systematic review or inclusion criteria referring to the systematic review, if available. We recorded the 2017 Journal Citation Reports (JCR) Journal Impact Factor (JIF) of a journal from the Web of Science. We hypothesised that manuscripts published in journals with a higher JIF would have a higher prevalence of SR definitions, due to the higher reporting standards.

For all data, one author (MKM) extracted data and the second author (LP) verified the extractions. Furthermore, one author (LP) categorised the definition elements and the second author verified the categorisations (MKM). Any discrepancies in opinion were resolved via discussion.

For the analysis of definitions from textbooks and Internet sources, we extracted the definitions verbatim and indicated the field from which the definition came from, such as medicine, psychology and social sciences. During the analyses of the textbooks, if the definition in the text was supplemented with a table, we treated this as one source of information and the extracted elements of the SR definition from both text and table. One author extracted data and the second author verified the extractions from the textbooks and Internet sources.

### Statistics

Descriptive statistics, including frequencies and percentages, were used to describe the categories of elements of a systematic review definition/inclusion criteria. We also analysed the frequency of each category by counting the categories of elements that were used in each source. If at least one element was used in a certain category, we considered that this category of elements was present in the information source. We expressed the JIF as the mean and standard deviation (M ± SD), we used a t-test to analyse the difference in the JIR between the information sources with and without a SR definition. For the analyses, we used the MedCalc statistical software, v 15.2.1 (©MedCalc Software bvba, Ostend, Belgium). The statistical significance was set at *P* < 0.05.

## Results

### Search results

After searching for OSRs and methodological studies, from the 347 identified full texts, we included 308 studies. We excluded 39 studies because 31 were duplicates and an additional eight manuscripts were excluded because they were written in Chinese or did not fit our inclusion criteria (commentaries, traditional narrative reviews, reviews of an unspecified type of reviews or analysed rapid reviews).

By searching Google Scholar we found 531 hits. Based on the limits we set, analysing 50 hits per search phrase, we analysed a total of 238 bibliographic records from Google Scholar. After removing the duplicates that we already had in the first cohort of the included studies, we included the remaining 200 manuscripts from this cohort of studies. Additionally, we analysed 27 textbooks. In total, we analysed 535 sources of information: 508 manuscripts from peer-reviewed journals and 27 from textbooks.

### Prevalence of definitions of SRs

Among the 535 analysed sources of information, 188 (35%) defined what they consider to be a systematic review, 62 (18%) had an inclusion criteria in the methods that allowed us to extract information about what the authors considered to be a systematic review and 59 (18%) had exclusion criteria that we used as well for determining the authors’ definition of a SR. Some sources of information had both a definition of a SR and/or inclusion/exclusion criteria; in total there were 226 sources of information from which we could extract information related to the authors’ definition of a SR.

Among the 508 manuscripts, we found a JIF for 401 manuscripts, of which 113 had a SR definition, and 288 did not. Journals that did not provide SR a definition had a higher JIF (4.4 ± 5.1) than those with a definition (3.7 ± 4.5), but this difference was not significant (*P* = 0.099).

### Organisations, databases and checklists used as a reference for SR definition

Many of the analysed sources explicitly mentioned relevant organisations, checklists and databases for defining what they considered to be a SR, some of the analysed sources of information only provided literature references to support their definitions or inclusion/exclusion criteria.

Explicit mentions of the names of the organisations, checklists, databases associated with a definition of SRs or criteria for the inclusion of SRs were found in 43 out of 535 (8%) analysed sources of information. Those were Cochrane (*N* = 24), the PRISMA statement (*N* = 13), criteria of Database of Reviews of Effect (DARE) (*N* = 5), National Institute for Health and Care Excellence (NICE) (*N* = 3), NHS Centre for Reviews and Dissemination (N = 3), Campbell collaboration (N = 2) National Health and Medical Research Council (*N* = 1), QUOROM (QUality Of Reporting of Meta-analyses) recommendations (N = 1), Guidelines from Agency for Healthcare Research and Quality (AHRQ) (N = 1), Institute of Medicine (IOM) (N = 1) and author Andy Oxman (N = 1), referred to as the “Oxman criteria“. Cochrane was mentioned most commonly, either as a reference to a whole organisation, the Cochrane Handbook for Systematic Reviews of Interventions or a specific Cochrane entity: the Dutch Cochrane Centre in one source of information. Details about the definitions and references provided in those 43 studies are shown in Additional file 1: Table S1. The most commonly used supporting references in those studies were the manuscripts by Moher et al. and Liberati et al. describing the PRISMA statement, the PRISMA-P checklist, and the Cochrane Handbook (Additional file 1: Table S1).

The most commonly used literature references that were used to support the statements provided in the definitions of SRs or the inclusion/exclusion criteria were also manuscripts describing the PRISMA statement and Cochrane Handbook (Additional file 1: Table S2).

### Elements of systematic review definitions

After analysing all the definitions of SRs and the inclusion/exclusion criteria for SRs, we extracted 188 individual elements of a SR definition; we categorised them into the following 14 categories: self-identification, indexing, aim/question, overall methods, search, identification of studies, selection of studies, study eligibility, data extraction, quality/bias/appraisal/validity, analysis/synthesis, describing included studies, reporting and unclear (Table 1).

**Table 1:** Categories and elements of systematic review definition found in health care literature; percentage calculated from 226 sources of information that had a SR definition, or inclusion/exclusion criteria that could be used for extracting individual elements of SR definition

Category	Element of definition	N (%)
Self-identified as a systematic review	Manuscript that identifies itself as a systematic review in title, abstract or in methods	30 (13)
Indexing	Indexed as SR	1 (0.4)
Aim/research question	Specific research question	66 (29)
	Clearly stated set of objectives	12 (5.3)
	Clearly formulated research question	11 (4.8)
	Focused research question	3 (1.3)
	Reported research question	2 (0.9)
	Clinical question including participants, interventions, controls, outcomes and study design (PICOS)	2 (0.9)
	Explicit clinical question	1 (0.4)
	Clearly stated topic of review	1 (0.4)
	Explicitly reported pre-defined objectives	1 (0.4)
	Stated goal implied a critical and comprehensive intent	1 (0.4)
	Clear statement of the topic	1 (0.4)
	Defined clinical topic	1 (0.4)
	Explicit statement of questions being addressed	1 (0.4)
Overall methods	Systematic methods	22 (9.7)
	Explicit methods	21 (9.2)
	Systematic method to minimize risk of bias	9 (4)
	Systematic approach, in an attempt to minimize biases and random errors, documented in the Materials and Methods section	8 (3.5)
	Explicit method to minimize risk of bias	7 (3.1)
	Reproducible methods	5 (2.2)
	Using a systematic approach	5 (2.2)
	Methods described in explicit detail	4 (1.8)
	Well-defined methods	2 (0.9)
	Overall methods defined study as systematic review	1 (0.4)
	Overall Conduct defined study as a systematic review	1 (0.4)
	Systematic review methodology on closer inspection of the methods section	1 (0.4)
	Specific methods	1 (0.4)
	Repeatable methods	1 (0.4)
	Rigorous methods	1 (0.4)
	Different components of the review process documented in the ‘methods section’	1 (0.4)
	Using methods to provide more reliable findings	1 (0.4)
	Using methods from which conclusions can be drawn	1 (0.4)
	Using methods based on which decisions can be made	1 (0.4)
	Exhaustive review of the literature	1 (0.4)
	Systematic approach	1 (0.4)
Search	Systematic search	29 (13)
	Reported search strategy	13 (5.8)
	Comprehensive search strategy	12 (5.3)
	Searched at least two databases/sources	10 (4.4)
	Exact search criteria reported	9 (4.0)
	Searched at least one database	9 (3.9)
	Reported search methods	7 (3.1)
	Attempt to collate all empirical evidence	7 (3.1)
	Reported all information sources	6 (2.6)
	Transparent search strategy	6 (2.6)
	Detailed and comprehensive search strategy (as identified by: naming of databases and years of searching and example or actual terms)	4 (1.8)
	Detailed and specific search strategy with key-words that enabled reproduction of the literature search	4 (1.8)
	Names of databases reported	4 (1.8)
	Explicit search criteria that are available to review	3 (1.3)
	Description of data sources and search dates	2 (0.4)
	Keywords searched	2 (0.9)
	Detailed search of the literature for relevant studies	2 (0.9)
	Explicit description of search strategy	2 (0.9)
	Adequate searching methods	2 (0.9)
	Replicable search method	2 (0.9)
	Reported search sources	1 (0.4)
	Description of sources	1 (0.4)
	Reported details of databases searched	1 (0.4)
	Reported dates of search	1 (0.4)
	Included relevant search strategy	1 (0.4)
	Adequate search strategy	1 (0.4)
	Appropriate search strategy	1 (0.4)
	Detailed search strategy	1 (0.4)
	Non-selective search strategy	1 (0.4)
	Explicit search strategy	1 (0.4)
	Prescriptive search strategy	1 (0.4)
	Reproducible search strategy	1 (0.4)
	Rigorous search process	1 (0.4)
	Explicitly reported search strategy details	1 (0.4)
	Thorough search of evidence	1 (0.4)
	Comprehensive search of evidence	1 (0.4)
	Reported search processes	1 (0.4)
	Extensive use of search string combinations	1 (0.4)
	Description of evidence retrieval methods	1 (0.4)
	Explicit and organized approach to searching	1 (0.4)
	Attempt to search all empirical evidence	1 (0.4)
	Adequately attempt to retrieve all relevant data	1 (0.4)
	Review trying to collect all available evidence	1 (0.4)
	Structured search of bibliographic and other databases	1 (0.4)
	Searched at least Medline	1 (0.4)
	Searched at least two databases (of which one is Medline)	1 (0.4)
Identification of studies	Explicit methods to identify relevant research	14 (6.2)
	Systematic methods of identification of studies	10 (4.4)
	Attempt to identify all empirical evidence	6 (2.6)
	Reported methods for identification of studies	2 (0.9)
	Transparent procedure to find relevant research	2 (0.9)
	Formal process of identifying literature	1 (0.4)
Selection of studies	Explicit methods to select relevant research	14 (6.2)
	Systematic methods of selection of studies	13 (5.8)
	Reported methods for selection of studies	6 (2.6)
	Transparent selection of studies	2 (0.9)
	Reproducible selection of studies	4 (1.8)
	Reproducible approach for selecting the studies	1 (0.4)
	Clear description of selection criteria	1 (0.4)
	Clear study selection criteria	1 (0.4)
	Relevant study selection criteria	1 (0.4)
	Detailed description of the studies’ selection process (number of articles included and excluded in each step)	1 (0.4)
Study eligibility	Reported inclusion and exclusion criteria	31 (14)
	Pre-defined/pre-specified eligibility criteria	20 (8.8)
	Outcome defined using a validated tool or diagnostic criteria	13 (5.8)
	Only Cochrane systematic reviews	12 (5.3)
	Reported inclusion criteria	6 (2.6)
	Explicitly reported inclusion and exclusion criteria	6 (2.6)
	Articles that meet PRISMA definition of a systematic review	5 (2.2)
	Definitions of the population(s), intervention(s), comparator(s) and outcome(s) of interest	2 (0.9)
	Inclusion/exclusion criteria that are relevant in terms of the PICO framework	3 (1.3)
	Reviews published in Database of Reviews of Effects (DARE)	2 (0.9)
	Reviews were judged to be systematic if they synthesized peer reviewed articles	1 (0.4)
	Studies meeting minimum methodological standards	1 (0.4)
	Reference to study designs	1 (0.4)
Data extraction	Systematic data collection	12 (5.3)
	Systematic methods to extract data	4 (1.8)
	Explicit methods to collect data	3 (1.3)
	Data extraction by 2 independent reviewers	2 (0.9)
	Reported data abstraction from trials	2 (0.9)
	Independent data extraction	1 (0.4)
	Explicit approach to extracting	1 (0.4)
	Organized approach to extracting	1 (0.4)
	Explicit methods to extract data	1 (0.4)
	Performed data extraction	1 (0.4)
	Extracting the information from the studies following a priori protocol	1 (0.4)
Quality, bias, appraisal, validity	Quality assessment of evidence	27 (12)
	Critical appraisal of the studies	25 (11)
	Risk of bias assessment	19 (8.4)
	Systematic methods to critically appraise relevant research	13 (5.8)
	Explicit methods to critically appraise relevant research	13 (5.8)
	Reported validity assessment	11 (4.9)
	Attempt to appraise all empirical evidence	6 (2.6)
	Full assessment of methodological quality of included studies	5 (2.2)
	Consideration of internal and external validity of the research	3 (1.3)
	Provided sufficient details about individual included studies to enable assessment of quality by a reader	2 (0.9)
	Reported at least one or more aspects of validity assessment of original studies	2 (0.9)
	Transparent procedures to evaluate relevant research	2 (0.9)
	Full report of methodological quality of included studies	1 (0.4)
	Transparent process to minimize risk of bias	1 (0.4)
	Explicit approach to critically evaluating studies	1 (0.4)
	Organized approach to critically evaluating empirical literature	1 (0.4)
	Systematic approach for assessing the studies	1 (0.4)
	Reproducible approach for assessing the studies	1 (0.4)
	Assessed methodological features of the included studies	1 (0.4)
	Adequate methods to appraise included studies	1 (0.4)
	Transparent methodological criteria are used to exclude papers that do not meet an explicit methodological benchmark	1 (0.4)
	Evaluate the retrieved studies using prospectively defined methodological criteria	1 (0.4)
Analysis, synthesis	Synthesis of results	34 (15)
	Presence of meta-analysis	19 (10)
	Systematic methods of analysis of studies	18 (8.0)
	Explicit methods to analyze data	17 (7.5)
	Systematic synthesis of findings	10 (4.4)
	Quantitative synthesis	9 (4.0)
	Synthesis of the included evidence, whether narrative or quantitative	7 (3.1)
	Attempt to synthesize all empirical evidence	6 (2.6)
	Systematic analysis of results	2 (0.9)
	Unbiased synthesis of study findings	2 (0.9)
	Transparent procedures to synthesize the results of relevant research	2 (0.9)
	Analyze results appropriately	1 (0.4)
	Systematic analysis	1 (0.4)
	Plausible analysis of data	1 (0.4)
	Plausible synthesis of data	1 (0.4)
	Summary of results	1 (0.4)
	Systematic analysis	1 (0.4)
	Meta-analysis or best evidence synthesis	1 (0.4)
	Formal analysis contained in the methods	1 (0.4)
	Makes judgement about research question	1 (0.4)
	Relying on statistical significance to make judgments about what works	1 (0.4)
	Transparent process of interpretation of the findings of the studies included in the review	1 (0.4)
	Rigorous conclusions about outcomes	1 (0.4)
Describing included studies	Systematic presentation of characteristics of included studies	4 (1.8)
	Systematic synthesis of characteristics of included studies	4 (1.8)
	Clearly identified all included studies	2 (0.9)
	Reported trial characteristics	1 (0.4)
	Systematic presentation of main information	1 (0.4)
	Described main characteristics of included studies	1 (0.4)
	Adequate methods to describe included studies	1 (0.4)
	Description of the number and nature of included studies	1 (0.4)
	Description of the types of primary studies included	1 (0.4)
	Accounted for identified studies	1 (0.4)
Reporting	Used PRISMA or predecessor guidelines for reporting	3 (2)
	Presented results appropriately	1 (0.4)
	Systematic presentation of findings	1 (0.4)
	Flow chart present	1 (0.4)
	Reported level of evidence for their recommendations	1 (0.4)
	Reported sufficient information to allow a level of evidence grading	1 (0.4)
	Published in a journal conforming to PRISMA standards	1 (0.4)
	A review that has methods and results section	1 (0.4)
Unclear	“It was apparent in the text that a systematic review had been undertaken”	4 (1.8)
	“Reviews were included if they were systematic”	1 (0.4)

Elements were sorted according to those categories (Table 1). The highest number of SR definition elements was found in categories related to searching (*N* = 51), analysis/synthesis (*N* = 23), overall methods (*N* = 22), quality/bias/appraisal/validity (N = 22) and aim/question (*N* = 13) (Table 1).

### Categories of systematic review elements

Among the 226 sources of information that had a SR definition or inclusion/exclusion criteria that could be used for extracting individual elements of a SR definition, 59 used only one category, 62 used two categories, while 105 used from three to ten categories of the SR definition elements. When we looked at the combinations that were used, none of the combinations of various categories was used more than ten times. The most commonly used combination of SR definition categories was used in nine of the manuscripts/books, and it used the following five categories: i) aim/research question, ii) search, iii) study eligibility, iv) quality, bias, appraisal, validity and v) analysis/synthesis. However, those nine manuscripts had different wording of the SR definition, as shown in Additional file 2: Table S3; they did not use one consistent definition.

The same five categories were the most commonly used SR definition categories in our sample of information sources, with the following frequencies: i) search (*N* = 122), ii) aim/research question (*N* = 93), iii) analysis/synthesis (*N* = 90), iv) study eligibility (*N* = 89) and v) quality, bias, appraisal, validity (*N* = 81).

## Discussion

We found that authors of manuscripts and textbooks use various definitions of systematic reviews; in 535 sources of information, we found 188 different elements of a SR definition. The most commonly used categories of SR definition elements were related to searching, analysis/synthesis, overall methods, quality/bias/appraisal/validity and aim/question. The most commonly used reference resources were the Cochrane and PRISMA statement [14, 15].

However, as our study showed, there is no uniformly used definition of a SR. We analysed various sources of information, including overviews of SRs and methodological studies about SRs because those studies included SRs and we expected that therefore they should provide a definition of a SR. Our expectations were not met; as we found that one-third of those information sources used an explicit definition of a SR. In another one-third of the information sources, we found either inclusion or exclusion criteria, from which we could deduce what they consider to be, or not to be, a SR.

Also, we found that journals that did not provide SR definition had a higher JIF than those with a definition, but this difference was not significant. This finding was not in line with our hypothesis, and it shows that in this respect journals with higher JIF did not have higher expectations from authors in terms of transparent reporting about what was considered to be a SR.

When extracting the elements of SR definitions, we tried to be as detailed as possible, to capture various terminology used in those definitions. We found many variations of similar concepts, but also many vague terms. Such vague terms were frequently reflected in the usage of the word systematic, such as: “systematic methods”, “systematic approach”, “systematic search”, “systematic synthesis”, “systematic analysis” and “systematic presentation”, without actually explaining what systematic means. We also found two expressions that were completely unclear about what the authors consider to be a SR, including “*Reviews were included if they were systematic*” and “*It was apparent in the text that a systematic review had been undertaken*”.

There were ten elements of a SR definition that used the type and number of sources that were searched in a SR, as an element of the SR definition. It has been suggested previously that a minimum number and types of sources should define SRs because searching only one database may not be universally considered a systematic search [16].

It could be argued that our categorisation was too detailed, as some of our categories of SR definition elements sound similar, for example, categories search, selection of studies, identification of studies and study eligibility. We left those categories as they were on purpose, because it may not be perfectly obvious what the difference between them is; for example, the term selection of studies in the Cochrane reviews is reserved for the description of the screening of abstracts and full texts, but it is unclear whether all authors use this term in the same context. Furthermore, it is unclear whether the identification of studies refers to searching, screening or eligibility, i.e. the inclusion/exclusion criteria. Because of this ambiguity, we chose to present more detailed categories.

The most commonly used individual five categories of the SR elements were also used as the most common combination of elements in the analysed sources of information, but only nine manuscripts used this combination of the five elements. Those five categories of elements are also included in the definition of SRs from the Cochrane Handbook [14].

In section 1.2.2 of the Cochrane Handbook, titled What is a systematic review?, the following definition can be found [quote]: “*A systematic review attempts to collate all empirical evidence that fits the pre-specified eligibility criteria in order to answer a specific research question. It uses explicit, systematic methods that are selected with a view to minimising bias, thus providing more reliable findings from which conclusions can be drawn and decisions made (Antman 1992, Oxman 1993). The key characteristics of a systematic review are: a clearly stated set of objectives with pre-defined eligibility criteria for the studies; an explicit, reproducible methodology; a systematic search that attempts to identify all the studies that would meet the eligibility criteria; an assessment of the validity of the findings of the included studies, for example through the assessment of the risk of bias; and a systematic presentation, and synthesis, of the characteristics and findings of the included studies”* [14].

Also, Cochrane was the most commonly mentioned organisation in the definitions of SRs; 13% of the manuscripts/textbooks mentioned Cochrane as a source of the SR definition. Therefore, one could argue that the Cochrane’s definition could be used as a formal definition of what a SR is. However, the Cochrane’s definition is also vague, as it is unclear what it means “*explicit, systematic methods” or “explicit, reproducible methodology”.* Someone can explicitly describe the methodology that is not adequate. This inadequate methodology may also be reproducible, but that does not mean that it is good. Furthermore, the Cochrane definition of a SR repeatedly uses the adjective “systematic”, without explaining what the meaning of systematic is.

Two references used in Cochrane’s definition of a SR are those of Antman et al. [17] and Oxman et al. [18]. We also analysed which references were used to support the definitions of SRs in the manuscripts and textbooks; we found that the authors most commonly referred to the PRISMA statement [15] and Cochrane Handbook. However, the definition of SRs from the PRISMA statement manuscripts also uses vague terms such as clearly, systematic and explicit, without going into details of what they entail [15].

The research community would benefit from having a very specific definition of a SR. The five most commonly used SR definition elements that we identified could be used to create a more elaborate and unambiguous definition of a SR. We believe that the international research community should create an unambiguous SR definition; we hope that this study will be a starting point in that direction. As a first step, we suggest starting with the following template:

A systematic review is a review that reports or includes the following:

i) research question
ii) sources that were searched, with a reproducible search strategy (naming of databases, naming of search platforms/engines, search date and complete search strategy)
iii) inclusion and exclusion criteria
iv) selection (screening) methods
v) critically appraises and reports the quality/risk of bias of the included studies
vi) information about data analysis and synthesis that allows the reproducibility of the results

Some of those elements are mentioned in the SR definition from the Cochrane Handbook [14], as shown above, but the Cochrane’s definition still leaves a lot of ambiguity in several aspects. Those elements should be more specific in future. For example, which details should the clinical question report, how many databases/sources should be searched to be considered systematic, whether key methodological aspects (screening of titles and abstracts, screening of full texts, data extraction and risk of bias assessment) should be done by two authors independently or done by one author and verified by another. The naming of the databases is important for ensuring transparency and reproducibility, which should be features of a systematic approach. Those and other considerations should be taken into account in further efforts to clarify what exactly makes a SR.

Information presented in this manuscript could help inform a consensus meeting or a similar gathering where interested SR researchers could contribute to standardising a SR definition. A similar approach was recently suggested for the definition of a predatory journal. Cobey et al. have conducted a scoping review in which they summarised the literature on predatory journals, described its epidemiological characteristics and extracted empirical descriptions of the potential characteristics of predatory journals. In their conclusions, they informed readers that the results will be shared with attendees that will attend a stakeholder meeting seeking to develop a standardised definition for what constitutes a predatory journal [19].

One limitation of our study could be the use of information sources published within a certain period of time. However, this type of work, which relies on analysis of published literature, usually suffers from a time lag. Each new update of the search results in new literature sources to analyse, and time lag appears again by the time analysis is completed.

Furthermore, in our approach, we analysed both expressions that appeared to be definitions of SRs and the characteristics of SRs eligible for inclusion. It may be considered that the inclusion criteria for SRs are not eligible elements to define what a SR is. However, we considered that the eligibility and inclusion criteria which describe SRs would be useful in our analysis, as we seldom found explicit statements about the definition of a SR. We consider that the range of descriptors we found indicates a very rich vocabulary used by authors who are defining or searching for SRs and that our approach is an adequate starting point towards building a future consensus definition of a systematic review. Likewise, it could be argued that we are mixing a definition of a SR with measures of the quality of a SR. However, in the absence of an existing definition, we believe that we should assess all the descriptors used for SRs and report them explicitly and transparently, then readers can see for themselves that some of those may overlap with quality descriptors. Also, for searching Google Scholar we used a limited number of phrases. Google used to include details for searching the advanced interface, which is no longer available but this search information could be available from other sites (mostly libraries) which we did not utilize.

Our analysis is also limited by the fact that we only focused on the definitions, while we acknowledge that some relevant information might also be found in the explanatory text to the definition, if available.

## Conclusion

The majority of manuscripts that include SRs actually do not provide a definition of what they consider to be a SR. The most commonly used reference sources of a SR definition use vague and ambiguous terms. We propose a new definition of a systematic review, which is open for further commenting and elaboration, with the aim of motivating the research community to create a more specific definition of this type of research.

## Supplementary information

**Additional file 1: Table S1.** Definitions of systematic reviews which explicitly quoted specific organizations/checklists/criteria. A table that contains definitions of systematic reviews, extracted from analyzed data sources, in which the authors hav explicitly quoted specific organizations, or checklists or criteria. **Table S2.** All references that were used to support definition of systematic review, or inclusion/exclusion criteria that could be used as a proxy for a definition. A list of references that autohrs of analyzed information sources have used in their manuscript to support either a definition of systematic review, or inclusion criteria, or exclusion criteria that could be used as a proxy indicator of a systematic review definition

**Additional file 2: Table S3.** Definitions of a systematic review from nine manuscripts with the most commonly used combination of categories. A file containing verbatim extracted definitions that were used in nine manuscripts that had the most commonly used combination of categories of systematic review definitions

## Data Availability

The datasets used and/or analyzed during the current study are available from the corresponding author on reasonable request.

## Abbreviations

AHRQ: Agency for Healthcare Research and Quality
DARE: Database of Reviews of Effect
EBM: Evidence-based medicine
IOM: Institute of Medicine
JCR: Journal Citation Reports
JIF: Journal Impact Factor
M ± SD: Mean and standard deviation
MA: Meta-analysis
NHS: National Health Service
NICE: National Institute for Health and Care Excellence
OSR: Overview of systematic reviews
PRISMA: Preferred Reporting Items for Systematic Reviews and Meta-Analyses
PRISMA-P: Preferred Reporting Items for Systematic Reviews and Meta-Analyses for Protocols
QUOROM: QUality Of Reporting of Meta-analyses
RCT: Randomized controlled trial
SR: Systematic review
