# Supplementary material for: Definition of a systematic review used in overviews of systematic reviews, meta-epidemiological studies and textbooks
Source: BMC Med Res Methodol. 2019 Nov 4;19:203. doi: 10.1186/s12874-019-0855-0 (PMC6829801; doi:10.1186/s12874-019-0855-0)
Supplement: Supplementary file 1 — Additional file 1: Table S1. Definitions of systematic reviews which explicitly quoted specific organizations/checklists/criteria. A table that contains definitions of systematic reviews, extracted from analyzed data sources, in which the authors hav explicitly quoted specific organizations, or checklists or criteria. Table S2. All references that were used to support definition of systematic review, or inclusion/exclusion criteria that could be used as a proxy for a definition. A list of references that autohrs of analyzed information sources have used in their manuscript to support either a definition of systematic review, or inclusion criteria, or exclusion criteria that could be used as a proxy indicator of a systematic review definition [file 12874_2019_855_MOESM1_ESM.docx]

**Supplementary table 1. Definitions of systematic reviews which explicitly quoted specific organizations/checklists/criteria**

| **Information source** | **Organization/checklist/author** | **Definition from inclusion/exclusion criteria (verbatim quotes)** | **Reference** |
| --- | --- | --- | --- |
| Bardus 2016 [[1](#_ENREF_1)] | NICE NHS Evidence Process and Methods Manual | "We included only ‘reliable systematic reviews’, defined in the NICE NHS Evidence Process and Methods Manual (27) as those published in a journal conforming to the PRISMA standards (28) or presenting inclusion/exclusion criteria, and confirming in the abstract that a synthesis of studies from two or more information sources had been undertaken.” | NICE 2015. [[2](#_ENREF_2)]  Moher 2009 [[3](#_ENREF_3)] |
| Chung 2015 [[4](#_ENREF_4)] | Definition from the Cochrane Handbook | “SR is defined as an “attempt to identify, appraise and synthesize all the empirical evidence that meets pre-specified eligibility criteria to answer a given research question”, in accordance to the Cochrane Handbook version 5.1.0.” | Higgins 2011 [[5](#_ENREF_5)] |
| Flodgren 2011 [[6](#_ENREF_6)] | DARE criteria | “Reviews rated as having an inadequate search strategy were excluded, defined by having searched less than two sources according to the DARE criteria” | Center for Reviews and Dissemination [[7](#_ENREF_7)] |
| Conway 2013 [[8](#_ENREF_8)] | PRISMA statement | “In accordance with the PRISMA statement, a systematic review was defined as a review with a clearly formulated question that used systematic and explicit methods to identify, select and critically appraise relevant research and to collect and analyze data from the studies that were included in the review (6, 35) The review had to describe a detailed search of the literature for relevant studies and synthesis of results” | Inglis 2011 [[9](#_ENREF_9)]  Chipps 2012 [[10](#_ENREF_10)] |
| Jaspers 2011 [[11](#_ENREF_11)] | Dutch Cochrane Center | "To determine whether a publication was a SR, we used the checklist for assessment of systematic reviews of the Dutch Cochrane Centre (6) For the initial screening of titles and abstracts, we considered a review to be systematic if at least (a) Medline had been searched, and (b) the methodological quality of the included studies had been assessed by the reviewer(s)" | Dutch Cochrane Centre 2009 [[12](#_ENREF_12)] |
| Kang 2012 [[13](#_ENREF_13)] | Cochrane Collaboration | We have adopted the definitions used by the Cochrane Collaboration: a systematic review is a review of a clearly formulated question, that which uses systematic and explicit methods to identify, select, and critically appraise relevant researches, and to collect and analyze data from the studies included in the review | Alderson 2004 [[14](#_ENREF_14)] |
| Kitsiou 2015 [[15](#_ENREF_15)] | Cochrane Collaboration and PRISMA | Following the definitions used by the Cochrane Collaboration and the Preferred Reporting Items for Systematic Reviews and Meta-Analyses (PRISMA) statement, a systematic review was defined as a review that attempts to search, identify, appraise, and collate all empirical evidence that fits pre-specified eligibility criteria to answer a clearly stated set of objectives or specific research question(s), using explicit and systematic methods with a view to minimizing risk of bias. | No reference |
| Mahtani 2013 [[16](#_ENREF_16)] | Cochrane Handbook | “We used the Cochrane Handbook for Systematic Reviews of Interventions for a definition of a systematic review, that is, ‘a systematic review attempts to identify, appraise and synthesize all the empirical evidence that meets prespecified eligibility criteria to answer a given research question’ (9)" | Higgins 2011 [[17](#_ENREF_17)] |
| McCall 2013 [[18](#_ENREF_18)] | Oxman criteria | "Included reviews must satisfy all Oxman criteria as follows: state a replicable search method; adequately attempt to retrieve all relevant data; collect the data in a systematic way; analyze and present the results appropriately; consider sources of bias and the quality of evidence [48]." | Oxman 1994 [[19](#_ENREF_19)] |
| Mickenautsch 2011 [[20](#_ENREF_20)] | QUOROM recommendations | "article exclusion criteria based on the QUOROM (QUality Of Reporting Meta-analysis) recommendations (23) for reporting of systematic review methods were used: reporting of the trial search processes; trial selection criteria; validity assessment of trials; data abstraction from trials; trial characteristics and quantitative synthesis." | Moher 1999 [[21](#_ENREF_21)] |
| Pollock 2014 [[22](#_ENREF_22)] | DARE | "This application of quality criteria ensures that systematic reviews in DARE have (1) reported inclusion or exclusion criteria, (2) employed an adequate search strategy and (3) synthesized included studies. In addition, to be included on DARE, a review must be considered to have assessed the quality of the included studies or provided sufficient details about individual included studies to enable assessment of quality by a reader." | No reference |
| Popovich 2012 [[23](#_ENREF_23)] | Cochrane, PRISMA | "We applied the Cochrane Collaboration definition of systematic reviews that is used by the Preferred Reporting Items for Systematic reviews and Meta-Analyses (PRISMA) statement: ‘‘The key characteristics of a systematic review are: (a) a clearly stated set of objectives with an explicit, reproducible methodology; (b) a systematic search that attempts to identify all studies that would meet the eligibility criteria; (c) an assessment of the validity of the findings of the included studies, for example through the assessment of risk of bias; and (d) systematic presentation, and synthesis, of the characteristics and findings of the included studies’’ [5]." | Moher 2009 [[24](#_ENREF_24)] |
| Remes 2016 [[25](#_ENREF_25)] | Cochrane, PRISMA | No specific definition:  “We defined a systematic review in accordance with the Cochrane Collaboration and the Preferred Reporting Items for Systematic Reviews and Meta-Analyses (PRISMA) Statement (Moher et al. 2009).” | Moher 2009 [[26](#_ENREF_26)] |
| Ryan 2014 [[27](#_ENREF_27)] | DARE | No specific definition:  Methodological aspects “according to the Centre for Reviews and Dissemination assessment of the review published as part of the DARE abstract” | No reference |
| Saez-Benito 2013 [[28](#_ENREF_28)] | PRISMA checklist | "Reviews were considered as systematic according to three criteria from the PRISMA check list [26]: (i) ‘State questions being addressed with reference to participants, interventions, comparisons, outcomes, and study design’; (ii) ‘Describe all information sources and date last searched’; (iii) ‘State studies selection process’." | Moher 2009 [[29](#_ENREF_29)] |
| Tafelski 2016 [[30](#_ENREF_30)] | PRISMA checklist | No specific definition:  “Systematic reviews should meet the preferred reporting items for systematic reviews and meta-analyses (PRISMA) criteria (12)” | Hutton 2015 [[31](#_ENREF_31)] |
| Tricco 2015 [[32](#_ENREF_32)] | Cochrane Collaboration | "We used the definition for a systematic review put forth by the Cochrane Collaboration, “A systematic review attempts to collate all empirical evidence that fits pre-specified eligibility criteria in order to answer a specific research question. It uses explicit, systematic methods that are selected with a view to minimizing bias, thus providing more reliable findings from which conclusions can be drawn and decisions made” [10]." | Higgins 2011 [[17](#_ENREF_17)] |
| Windsor 2012 [[33](#_ENREF_33)] | Cochrane Collaboration and PRISMA | "We used the definition of systematic reviews used by the CR Collaboration and in the PRISMA statement: ‘A systematic review is a review of a clearly formulated question that uses systematic and explicit methods to identify, select and critically appraise relevant research, and to collect and analyze data from the studies that are included in the review. Statistical methods (meta-analysis) may or may not be used to analyze and summarize the results of the included studies. Meta-analysis refers to the use of statistical techniques in a systematic review to integrate the results of included studies’ (Egger et al., 2001)." | Egger 2001 [[34](#_ENREF_34)] |
| Bidonde 2014 [[35](#_ENREF_35)] | Cochrane Collaboration | Although there is no standard definition of a systematic review (SR) [16] we followed the key characteristics of a systematic review as stated by the Cochrane Collaboration [17]. | Moher 2007 [[36](#_ENREF_36)]  Higgins 2011 [[17](#_ENREF_17)] |
| Chen 2016 [[37](#_ENREF_37)] | Cochrane Handbook | A systematic review, defined by Handbook, is tried to collect all the available evidences that meet prescribed eligibility criteria so as to answer a specific research question. It uses specific and systematic methods, aimed at minimizing bias, to provide more reliable findings, from which, conclusions can be drawn and decisions made. Many systematic reviews contain meta-analyses. Meta-analysis is a statistical method used to synthesize the results of several independent studies [13]. | Higgins 2011 [[17](#_ENREF_17)] |
| Howells 2016 [[38](#_ENREF_38)] | Centre for Reviews and Dissemination Database of Abstracts of Reviews of Effects criteria | Studies were classed as systematic reviews if they met the Centre for Reviews and Dissemination Database of Abstracts of Reviews of Effects criteria (see table 2) 22 and included a clear statement of the clinical topic, description of evidence retrieval methods and sources, and at least one study that met minimum methodological standards for inclusion — as per additional guidance. 21, 23 | Centre for Reviews and Dissemination [[7](#_ENREF_7)]  Wilczynski NL 2007 [[39](#_ENREF_39)]  Montori 2005 [[40](#_ENREF_40)] |
| Kumar 2013 [[41](#_ENREF_41)] | PRISMA | No definition  „Articles that did not meet the PRISMA (13) definition of a systematic review or meta-analysis were excluded.” | Liberati 2009 [[42](#_ENREF_42)] |
| Macaya Pascual 2016 [[43](#_ENREF_43)] | DARE | No definition  „used an appropriate search strategy (articles indexed by DARE, Database of Abstracts of Reviews of Effects) (12) “ | Centre for R, Dissemination 2009 [[7](#_ENREF_7)] |
| Rotta 2015 [[44](#_ENREF_44)] | PRISMA statement checklist | A study was considered to be a systematic review if it satisfactorily fulfilled the following three items of the PRISMA Statement checklist: (1) item 4: a clear description of the clinical question to be answered by the systematic review, including participants, interventions, controls, outcomes and study design (PICOS); (2) item 7: a description of all data sources used to retrieve the literature and the search period considered; and (3) item 9: a detailed description of the studies’ selection process (number of articles included and excluded in each step) [23]. | Moher 2010 [[45](#_ENREF_45)] |
| Wu 2015 [[46](#_ENREF_46)] | Cochrane Handbook | The Cochrane handbook version 5.1.0.10 states that an SR should aim to ‘identify, appraise and synthesize all the empirical evidence that meets pre-specified eligibility criteria to answer a given research question’. Following this definition, we judged a publication as an SR if it sought to answer an explicit clinical question by examining evidence from at least two electronic databases. | Higgins 2011 [[17](#_ENREF_17)] |
| Korzeniewski 2008 [[47](#_ENREF_47)] | National Health and Medical Research Council Guidelines | "(…) is consistent also with the definition of a “systematic” review found in National Health and Medical Research Council Guidelines (p 2 and p 12). A systematic review (1) makes a thorough attempt to identify all relevant research, (2) makes judgments about the quality of the literature, (3) systematically synthesizes the findings of studies of acceptable quality, and (4) makes judgments about the research question." | National Health and Medical Research Council [[48](#_ENREF_48)]  National Health and Medical Research Council [[49](#_ENREF_49)] |
| Willis 2011 [[50](#_ENREF_50)] | Cochrane Handbook for Systematic Reviews  PRISMA | "The definition of a systematic review is open to interpretation. Chalmers and Altman described a systematic review as a review, which had been prepared using a ‘systematic approach to minimizing biases and random errors’, with the different components of the process. being documented in the ‘methods section’ [34]. The Cochrane Handbook for Systematic Reviews on Interventions states that systematic reviews possess a number of ‘key characteristics’ [35]. The eligibility criteria used here were in line with these key characteristics and also coincide with those items of PRISMA that help define a systematic review." | Chalmers 1995 [[51](#_ENREF_51)]  Higgins 2011 [[17](#_ENREF_17)] |
| Kastner 2012 [[52](#_ENREF_52)] | Cochrane Collaboration | The definition of a systematic review according to the Cochrane Collaboration is “A review of clearly formulated questions that uses systematic and explicit methods to identify, select, and critically appraise relevant research, and to collect and analyze data from the studies that are included in the review. Statistical methods (meta-analysis) may or may not be used to analyze and summarize the results of the included studies” | No reference |
| Evans 2015 [[53](#_ENREF_53)] | Campbell collaboration  Cochrane | "There is no single definition of a systematic review, but in considering how systematic each of the reviews we examine is, we turn to guidance from two main registries of systematic reviews, the Campbell Collaboration and Cochrane. The Campbell Collaboration (2015) defines a systematic review as one that “uses transparent procedures to find, evaluate and synthesize the results of relevant research. Procedures are explicitly defined in advance, in order to ensure that the exercise is transparent and can be replicated.” Campbell also describes screening studies for quality and peer review as important elements of systematic reviews. They provide four specific criteria that a review must have in order to be considered systematic: (a) clear inclusion/exclusion criteria; (b) an explicit search strategy; (c) systematic coding and analysis of included studies; and (d) meta-analysis (where possible). Cochrane provides less-specific guidance on what makes a review systematic, but its description of its own reviews is highly correlated with that of the Campbell Collaboration (Cochrane 2015). (…)" | Campbell Collaboration 2015 [[54](#_ENREF_54)]  Cochrane 2015 [[55](#_ENREF_55)] |
| Haase 2011 [[56](#_ENREF_56)] | Cochrane | "Traditional narrative reviews often fail to use scientific method and are therefore subject to significant bias. In an effort to reduce this bias and improve the scientific rigor of reviews, the systematic review was developed in the 1990s. Chalmers and Altman defined the systematic review as “a review that has been prepared using a systematic approach to minimizing biases and random errors which is documented in a materials and methods section.” The Cochrane Handbook’s definition of a systematic review is one that “attempts to collate all empirical evidence that fits prespecified eligibility criteria to answer a specific research question. It uses explicit systematic methods that are selected with a view to minimizing bias, thus providing more reliable findings from which conclusions can be drawn and decisions made.” As might be inferred by the above definitions, systematic reviews are conducted according to structured guidelines to maintain their scientific integrity and minimize bias." | Chalmers 1995 [[51](#_ENREF_51)] |
| Foisy 2011 [[57](#_ENREF_57)] | Cochrane collaboration | To be included, reviews had to fulfill The Cochrane Collaboration’s definition of a systematic review (i.e. ‘reviews of clearly formulated questions that use systemic and explicit methods to identify, select and critically appraise relevant research, and to collect and analyze data from the studies included in the reviews’ | Waters 2006 [[58](#_ENREF_58)] |
| Kapadia 2016 [[59](#_ENREF_59)] | PRISMA | PRISMA-P-C and PRISMA-C have adopted the same definition of a ‘systematic review’ and ‘protocol’ as PRISMA-P5 and PRISMA.  A systematic review collates all relevant evidence that fits prespecified eligibility criteria to answer a specific research question. It uses explicit, systematic methods to minimize bias in the identification, selection, synthesis and summary of relevant studies. A protocol is a document that presents an explicit plan for a systematic review and details the rationale and a priori methodological and analytical approaches for the review. | Moher 2015 [[60](#_ENREF_60)]  Moher 2009 [[3](#_ENREF_3)] |
| Petrosino 2007 [[61](#_ENREF_61)] | NHS Centre for Reviews and Dissemination | Using the term "systematic review" seems to get us out of some of those quandaries but may lead us into others. One general rule used to define a systematic review is that it will usually include a "methodology and results" section. But a review could use systematic methods to summarize evaluation studies, and then rely on "statistical significance" to make judgments about "what works." This definition would classify such a review as systematic even though there are empirical reasons undermining its conclusions. The definition of systematic review created by Khan and his colleagues at the NHS Centre for Reviews and Dissemination (200 1:1) would also treat vote counting, a formerly popular method of summarizing studies within a review, in similar fashion: A review of the evidence on a clear formulated question that uses systematic and explicit methods to identify, select and critically appraise relevant primary research, and to extract and analyze data from the studies included in the review. Given the definitional problems, we prefer to think of systematic reviews as ranging on a continuum of quality. | Khan 2001 [[62](#_ENREF_62)] |
| Robertson 2015 [[63](#_ENREF_63)] | Cochrane Handbook | No definition.  "The following review process measure was devised based on the Cochrane Handbook definition of systematic review and a systematic review quality assessment tool (Shea et al., 2007)." | Shea 2007 [[64](#_ENREF_64)] |
| Kaltenthaler 2011 [[65](#_ENREF_65)] | NICE  Centre for Review and Dissemination (CRD) | No definition  "The NICE Reference Case requires that evidence to inform parameters of clinical effectiveness should be identified by systematic review. In its specification or definition of ‘systematic review’ for these parameters, the NICE Methods Guide refers to the systematic review methods of the Centre for Review and Dissemination (CRD)." | No reference |
| Lee 2016 [[66](#_ENREF_66)] | AHRQ  Cochrane  IOM | We defined systematic review as (a) using a set of keywords in (b) two or more databases with (c) independent coders assessing all identified records for inclusion or exclusion. Guidelines from Agency for Healthcare Research and Quality (AHRQ) (recommendation 7.6.6) [13], Cochrane (recommendation 7.2.4) [14], and the U.S. Institute of Medicine (IOM; recommendation 3.3.3) [15] all recommend dual independent coding for inclusion to reduce error and increase confidence in the findings. | No reference |
| Bhurke 2015 [[67](#_ENREF_67)] | Cochrane  NICE | By extending Jones et al. paper to include a clear definition of ‘systematic review’ to determine which reviews should be included may have inadvertently resulted in differences between our study and theirs However, by predefining a description for each area of use these definitions can now be piloted for similar research. (…) We defined a systematic review as previously existing if we found, relating to the trial research question, one or more of the following:  -a Cochrane systematic review  -other reviews if systematic review was mentioned in the title and methods stated a systematic search was conducted  -a National Institute for Health and Care Excellence (NICE) Technology Appraisal Guidance (TA) which include the Technology Assessment Report (TAR) based on the review of clinical and economic evidence | No reference |
| Tsakalerou 2015 [[68](#_ENREF_68)] | PRISMA | PRISMA adopts the definition of systematic review of a clearly formulated question as the systematic and explicit use of methods to identify, select, and critically appraise the data from the studies included in the review. | Moher 2009 [[59](#_ENREF_59)] |
| Hansen 2013 [[69](#_ENREF_69)] | Cochrane  Campbell Collaboration | We also develop an operational definition of ‘systematic review’, which is sufficiently broad to encompass both traditional systematic reviews, such as those undertaken under the auspices of the Cochrane and Campbell Collaborations, and more unconventional ones, such as realist reviews, employed within the social sciences.  There is no shortage of definitions of systematic reviews. For some, it is simply a process of offering accountable, replicable and updateable piece of research to the involved users (EPPI-Centre, 2009a). For others, it is more narrowly defined. The Campbell Collaboration defines a systematic review as ‘a transparent procedure to find, evaluate and synthesize the results of relevant research’. According to the Cochrane Collaboration, a systematic review is a ‘high-level overview of primary research on a particular research question that tries to identify, select, synthesize and appraise all high-quality research evidence relevant to that question in order to answer it’. As such, systematic reviews seek, summarize and interpret primary studies while attempting to provide unbiased research evidence on a given topic. They need to be rigorous in their approach to summarizing and interpreting the evidence. If not, they are ‘little more than ... subjective commentaries on the state of the science’ (Weed, 2013, p. 280). (…) | EPPI-Centre, 2009 [[70](#_ENREF_70)]  Campbell Collaboration [[71](#_ENREF_71)]  Cochrane Collaboration [[72](#_ENREF_72)]  Weed 2013 [[73](#_ENREF_73)] |
| Winter 2009 [[74](#_ENREF_74)] | The NHS Centre for Reviews and Dissemination | The NHS Centre for Reviews and Dissemination (2001) has defined a systematic review as: ‘A review of the evidence of clearly formulated questions that uses systematic and explicit methods to identify, select and critically appraise relevant primary research, and to extract and analyze data from the studies that are included in the review. Statistical methods (meta-analysis) may or may not be used.’ (Stage I, Phase 0, p4) | University of York 2001 [[75](#_ENREF_75)] |
| Soares 2014 [[76](#_ENREF_76)] | Cochrane Collaboration | Founded on the concept of the Cochrane Collaboration, systematic review was defined as the method of choice for evidence-based practice, which combines findings from multiple studies focused on a specific problem. | No reference |
| van der Linde 2012 [[77](#_ENREF_77)] | Cochrane Collaboration  PRISMA | A systematic review was defined as used by the Cochrane Collaboration and the Preferred Reporting Items for Systematic Reviews and Meta-Analyses (PRISMA) Statement: “A review of a clearly formulated question that uses systematic and explicit methods to identify, select, and critically appraise relevant research, and to collect and analyze data from the studies that are included in the review. Statistical methods (meta-analysis) may or may not be used to analyze and summarize the results of the included studies” | Moher 2009 [[26](#_ENREF_26)] |
| Aveyard 2014 [[78](#_ENREF_78)] | Cochrane Collaboration | In the most detailed form, a systematic review strives to identify comprehensively and track down all the available literature on a topic, while describing a clear, comprehensive methodology. Systematic reviews have been identified as "concise summaries of the best available evidence that address sharply defined clinical questions" (Murlow et al. 1997, p 389). The most well-known method for conducting a systematic review is produced by the Cochrane Collaboration ([www.cochrane.org](http://www.cochrane.org)).  One of the main features of a systematic review is that reviews follow a strict protocol to ensure that the review process undertaken is systematic by using explicit and rigorous methods to identify, critically appraise and synthesize relevant studies in order to answer a predefined question. The reviewers then develop a comprehensive search strategy, leaving no stone unturned in the search for relevant literature, and do not regard the process complete until the search is exhausted. For example, reviewers search for unpublished research and might talk to researchers about unpublished data of articles not accepted for publication, in addition to published data on the topic in question.  Reviewers then develop inclusion and exclusion criteria in order to assess which information they retrieve should be incorporated into the review and to ensure that only those papers that are relevant to the question(s) addressed by the literature review are included. The reviews than critique the selected papers according to predetermined criteria in order to assess the quality or validity of the research identified. studies that do not meet the inclusion criteria are excluded from the review. This is to ensure that only high-quality papers that are relevant to the literature review questions are included. This process is usually undertaken by two reviewers that collaborate to ensure there is agreement about which studies are included. Finally, the findings of all papers that are identified and incorporated for the review are pulled together and combined using a systematic approach. This enables new insights to be drawn from the summary of the papers that were not available before. | Mulrow 1997 [[79](#_ENREF_79)]  Cochrane Collaboration [[72](#_ENREF_72)] |

**Supplementary table 2. All references that were used to support definition of systematic review, or inclusion/exclusion criteria that could be used as a proxy for a definition**

| **Information source** | **Reference** | **N** |
| --- | --- | --- |
| Bardus 2016 [[1](#_ENREF_1)] | National Institute for Health and Care Excellence 2015 [[2](#_ENREF_2)] | 1 |
| Bardus 2016 [[1](#_ENREF_1)]  Ilomaki 2015 [[80](#_ENREF_80)]  Oestergaard 2011 [[81](#_ENREF_81)]  Akram 2014 [[82](#_ENREF_82)]  Kapadia 2016 [[59](#_ENREF_59)] | Moher 2009 [[3](#_ENREF_3)] | 5 |
| Borge 2014 [[83](#_ENREF_83)] | Shea 2009 [[84](#_ENREF_84)] | 1 |
| Chung 2015 [[4](#_ENREF_4)]  Wu 2015 [[46](#_ENREF_46)]  Mahtani 2013 [[16](#_ENREF_16)]  Rogante 2015 [[85](#_ENREF_85)]  Tricco 2015 [[32](#_ENREF_32)]  Chen 2016 [[37](#_ENREF_37)]  Shikora 2015 [[86](#_ENREF_86)]  Thomas 2012 [[87](#_ENREF_87)] | Higgins 2011 [[17](#_ENREF_17)] | 8 |
| Faggion 2012 [[88](#_ENREF_88)] | Vigna-Taglianti 2006 [[89](#_ENREF_89)] | 1 |
| Fleming 2013 [[90](#_ENREF_90)] | Sequeira-Byron 2011 [[91](#_ENREF_91)] | 1 |
| Fleming 2013 [[90](#_ENREF_90)]  Sequeira-Byron 2011 [[91](#_ENREF_91)] | Sander 2006 [[92](#_ENREF_92)] | 2 |
| Conway 2013 [[8](#_ENREF_8)] | Inglis 2011 [[9](#_ENREF_9)] | 1 |
| Conway 2013 [[8](#_ENREF_8)] | Chipps 2012 [[10](#_ENREF_10)] | 1 |
| Hunter 2016 [[93](#_ENREF_93)]  Momeni 2015 [[94](#_ENREF_94)]  Momeni 2013 [[95](#_ENREF_95)] | Lundh 2009 [[96](#_ENREF_96)] | 3 |
| Jaspers 2011 [[11](#_ENREF_11)] | Dutch Cochrane Centre 2009 [[97](#_ENREF_97)] | 1 |
| Kang 2012 [[13](#_ENREF_13)] | Alderson 2004 [[98](#_ENREF_98)] | 1 |
| Laver 2016 [[99](#_ENREF_99)]  Winter 2009 [[74](#_ENREF_74)] | University of York 2001 [[75](#_ENREF_75)] | 2 |
| Martinez-Gonzales 2014 [[100](#_ENREF_100)]  Windsor 2012 [[33](#_ENREF_33)] | Egger 2001 [[34](#_ENREF_34)] | 2 |
| McCall 2013 [[18](#_ENREF_18)] | Oxman 1994 [[19](#_ENREF_19)] | 1 |
| Mickenautsch 2011 [[20](#_ENREF_20)] | Moher 1999 [[21](#_ENREF_21)] | 1 |
| O’Connell 2013 [[101](#_ENREF_101)]  Robertson 2015 [[63](#_ENREF_63)] | Shea 2007 [[64](#_ENREF_64)] | 2 |
| Plaszewski 2014 [[102](#_ENREF_102)]  Plaszewski 2014 [[103](#_ENREF_103)] | Cochrane Collaboration 2005 [[104](#_ENREF_104)] | 2 |
| Popovich 2012 [[23](#_ENREF_23)] | Moher 2009 [[24](#_ENREF_24)] | 1 |
| Remes 2016 [[25](#_ENREF_25)]  van der Linde 2012 [[77](#_ENREF_77)] | Moher 2009 [[26](#_ENREF_26)] | 2 |
| Saez-Benito 2013 [[28](#_ENREF_28)]  Sekhon 2017 [[105](#_ENREF_105)] | Moher 2009 [[29](#_ENREF_29)] | 2 |
| Savard 2011 [[106](#_ENREF_106)] | Whitlock 2008 [[107](#_ENREF_107)] | 1 |
| Tafelski 2016 [[30](#_ENREF_30)] | Hutton 2015 [[31](#_ENREF_31)] | 1 |
| Yucel 2016 [[108](#_ENREF_108)] | Liberati 2009 [[109](#_ENREF_109)] | 1 |
| Bidonde 2014 [[35](#_ENREF_35)]  Page 2013 [[110](#_ENREF_110)]  Beller 2011 [[111](#_ENREF_111)]  Beller 2013 [[112](#_ENREF_112)]  Faggion 2014 [[113](#_ENREF_113)] | Moher 2007 [[36](#_ENREF_36)] | 5 |
| Bidonde 2014 [[35](#_ENREF_35)] | Green 2011 [[17](#_ENREF_17)] | 1 |
| Howells 2016 [[38](#_ENREF_38)] | Centre for Reviews and Dissemination [[7](#_ENREF_7)] | 1 |
| Howells 2016 [[38](#_ENREF_38)] | Wilczynski 2007 [[39](#_ENREF_39)] | 1 |
| Howells 2016 [[38](#_ENREF_38)] | Montori 2005 [[40](#_ENREF_40)] | 1 |
| Lu 2014 [[114](#_ENREF_114)] | Abalos 2001 [[115](#_ENREF_115)] | 1 |
| Lu 2014 [[114](#_ENREF_114)] | Ernst 2011 [[116](#_ENREF_116)] | 1 |
| Macaya Pascual 2016 [[43](#_ENREF_43)] | Centre for Reviews and Dissemination [[7](#_ENREF_7)] | 1 |
| Rotta 2015 [[44](#_ENREF_44)] | Moher 2010 [[45](#_ENREF_45)] | 1 |
| Wu 2015 [[46](#_ENREF_46)] | Smith 2011 [[117](#_ENREF_117)] | 1 |
| Korzeniewski 2008 [[47](#_ENREF_47)] | National Health and Medical Research Council 1999 [[49](#_ENREF_49)] | 1 |
| Willis 2011 [[50](#_ENREF_50)]  Haase 2011 [[56](#_ENREF_56)]  Bowling 2014 [[118](#_ENREF_118)] | Chalmers 1995 [[51](#_ENREF_51)] | 3 |
| Willis 2011 [[50](#_ENREF_50)] | Higgins 2011 [[17](#_ENREF_17)] | 1 |
| Bambra 2011 [[119](#_ENREF_119)] | Oakley 1998 [[120](#_ENREF_120)] | 1 |
| Evans 2015 [[53](#_ENREF_53)] | Campbell Collaboration 2015 [[71](#_ENREF_71)] | 1 |
| Evans 2015 [[53](#_ENREF_53)] | Cochrane 2015 [[55](#_ENREF_55)] | 1 |
| Jesson 2006 [[121](#_ENREF_121)] | Khan 2003 [[122](#_ENREF_122)] | 1 |
| Mueller 2014 [[123](#_ENREF_123)] | Peters 2006 [[124](#_ENREF_124)] | 1 |
| Mueller 2014 [[123](#_ENREF_123)] | Korevaar 2011 [[125](#_ENREF_125)] | 1 |
| Potting 2009 [[126](#_ENREF_126)] | Cochrane 2007 [[127](#_ENREF_127)]  . | 1 |
| Iwarsson 2017 [[128](#_ENREF_128)] | Cochrane Library [[129](#_ENREF_129)] | 1 |
| Foisy 2011 [[57](#_ENREF_57)] | Waters 2006 [[58](#_ENREF_58)] | 1 |
| Neto 2007 [[130](#_ENREF_130)]  Baldassarre 2008 [[131](#_ENREF_131)] | Kitchenham 2004 [[132](#_ENREF_132)] | 2 |
| Kapadia 2016 [[59](#_ENREF_59)] | Moher 2015 [[60](#_ENREF_60)] | 1 |
| Lichtner 2014 [[133](#_ENREF_133)] | Zwakhalen 2006 [[134](#_ENREF_134)] | 1 |
| Lichtner 2014 [[133](#_ENREF_133)] | Liberati 2009 [[135](#_ENREF_135)] | 1 |
| Pope 2006 [[136](#_ENREF_136)] | The Jenner Institute [[137](#_ENREF_137)] | 1 |
| Petrosino 2007 [[61](#_ENREF_61)] | Khan 2001 [[62](#_ENREF_62)] | 1 |
| Daigneault 2014 [[138](#_ENREF_138)] | Petticrew 2006 [[139](#_ENREF_139)] | 1 |
| Ranson 2010 [[140](#_ENREF_140)] | Lavis 2007 [[141](#_ENREF_141)] | 1 |
| Pollock 2014 [[22](#_ENREF_22)] | Evidence-informed policy and practice (EPPI) centre 2016 [[70](#_ENREF_70)] | 1 |
| Marinho 2003 [[142](#_ENREF_142)]  Aveyard 2014 [[78](#_ENREF_78)] | Mulrow 1997 [[143](#_ENREF_143)] | 2 |
| Marinho 2003 [[142](#_ENREF_142)] | University of York 1996 [[144](#_ENREF_144)] | 1 |
| Newcomer 2015 [[145](#_ENREF_145)] | Chalmers 2002 [[146](#_ENREF_146)] | 1 |
| Tsakalerou 2015 [[68](#_ENREF_68)] | Moher 2009 [[147](#_ENREF_147)] | 1 |
| Sanchez-Puerta 2016 [[148](#_ENREF_148)] | Waddington 2012 [[149](#_ENREF_149)] | 1 |
| Baude 2017 [[150](#_ENREF_150)] | Garg 2008 [[151](#_ENREF_151)] | 1 |
| Franco [[152](#_ENREF_152)] | Bambra 2011 [[119](#_ENREF_119)] | 1 |
| Hansen 2013 [[69](#_ENREF_69)] | EPPI-Centre 2009 [[70](#_ENREF_70)]  Campbell Collaboration,  Cochrane Collaboration,  Weed, 2013, p. 280 | 1 |
| Best 2012 [[153](#_ENREF_153)] | Greenhalgh 2004 [[154](#_ENREF_154)] | 1 |
| Maitland 2015 [[155](#_ENREF_155)] | Klassen 1998 [[156](#_ENREF_156)] | 1 |
| Svejvig 2015 [[157](#_ENREF_157)]  Shukla 2013 [[158](#_ENREF_158)]  Bastos 2017 [[159](#_ENREF_159)] | Tranfield 2003 [[160](#_ENREF_160)] | 3 |
| Neale 2009 [[161](#_ENREF_161)] | Oxman 1988 [[162](#_ENREF_162)] | 1 |
| Sjöholm 2013 [[163](#_ENREF_163)]  Sjöholm 2013 [[164](#_ENREF_164)] | Jesson 2011 [[165](#_ENREF_165)] | 2 |
| Matney 2015 [[166](#_ENREF_166)] | Gough 2012 [[167](#_ENREF_167)] | 1 |
| Matney 2015 [[166](#_ENREF_166)] | Hanson-Abromeit 2014 [[168](#_ENREF_168)] | 1 |
| Matney 2015 [[166](#_ENREF_166)] | Porta 2008 [[169](#_ENREF_169)] | 1 |
| Matney 2015 [[166](#_ENREF_166)] | Khan 2011 [[170](#_ENREF_170)] | 1 |
| Marshall 2016 [[171](#_ENREF_171)]  Marshall 2018 [[172](#_ENREF_172)] | Mulrow 1994 [[173](#_ENREF_173)] | 2 |
| Marshall 2016 [[171](#_ENREF_171)]  Marshall 2018 [[172](#_ENREF_172)] | Cook 1997 [[174](#_ENREF_174)] | 2 |
| Marshall 2016 [[171](#_ENREF_171)]  Marshall 2018 [[172](#_ENREF_172)] | Ba 2007 [[175](#_ENREF_175)] | 2 |
| Marshall 2016 [[171](#_ENREF_171)] | Haddaway 2014 [[176](#_ENREF_176)] | 1 |
| Bastos 2017 [[159](#_ENREF_159)] | Thomé 2016 [[177](#_ENREF_177)] | 1 |
| Aveyard 2014 [[78](#_ENREF_78)] | Cochrane Collaboration [[72](#_ENREF_72)] | 1 |
| Hall 2017 [[178](#_ENREF_178)] | Littell 2008 [[179](#_ENREF_179)] | 1 |
| Higgins 2008 [[180](#_ENREF_180)] | Antman 1992 [[181](#_ENREF_181)] | 1 |
| Higgins 2008 [[180](#_ENREF_180)] | Oxman 1993 [[182](#_ENREF_182)] | 1 |
| Holly 2012 [[183](#_ENREF_183)] | Centre for Reviews and Dissemination 2009 [[184](#_ENREF_184)] | 1 |
| Bowling 2014 [[118](#_ENREF_118)] | O'Rourke 2005 [[185](#_ENREF_185)] | 1 |
| Sharma 2015 [[186](#_ENREF_186)] | Crowther 2007 [[187](#_ENREF_187)] | 1 |
| Sharma 2015 [[186](#_ENREF_186)] | Khan 2003 [[188](#_ENREF_188)] | 1 |
| Sharma 2015 [[186](#_ENREF_186)] | Evidence-Based Behavioral Practice 2013 [[189](#_ENREF_189)] | 1 |

**References**

[1] Bardus M, Smith JR, Samaha L, Abraham C. Mobile and Web 2.0 interventions for weight management: an overview of review evidence and its methodological quality. European journal of public health. 2016;26:602-10.

[2] National Institute for H, Care E. NHS Evidence Process and Methods Manual. National Institute for Health and Care Excellence (NICE); 2015.

[3] Moher D, Liberati A, Tetzlaff J, Altman DG, Group P. Preferred reporting items for systematic reviews and meta-analyses: the PRISMA statement. PLoS medicine. 2009;6:e1000097.

[4] Chung VC, Wu X, Hui EP, Ziea ET, Ng BF, Ho RS, et al. Effectiveness of Chinese herbal medicine for cancer palliative care: overview of systematic reviews with meta-analyses. Scientific reports. 2015;5:18111.

[5] Higgins JPT, Green S, Cochrane C. Cochrane handbook for systematic reviews of interventions. 2011.

[6] Flodgren G, Eccles MP, Shepperd S, Scott A, Parmelli E, Beyer FR. An overview of reviews evaluating the effectiveness of financial incentives in changing healthcare professional behaviours and patient outcomes. The Cochrane database of systematic reviews. 2011:CD009255.

[7] Centre for Reviews and Dissemination. https://[www.york.ac.uk/crd/;](http://www.york.ac.uk/crd/;) [accessed 30 August 2018].

[8] Conway A, Inglis SC, Chang AM, Horton-Breshears M, Cleland JG, Clark RA. Not all systematic reviews are systematic: a meta-review of the quality of systematic reviews for non-invasive remote monitoring in heart failure. Journal of telemedicine and telecare. 2013;19:326-37.

[9] Inglis SC, Clark RA, McAlister FA, Stewart S, Cleland JG. Which components of heart failure programmes are effective? A systematic review and meta-analysis of the outcomes of structured telephone support or telemonitoring as the primary component of chronic heart failure management in 8323 patients: Abridged Cochrane Review. European journal of heart failure. 2011;13:1028-40.

[10] Chipps J, Brysiewicz P, Mars M. Effectiveness and feasibility of telepsychiatry in resource constrained environments? A systematic review of the evidence. African journal of psychiatry. 2012;15:235-43.

[11] Jaspers MW, Smeulers M, Vermeulen H, Peute LW. Effects of clinical decision-support systems on practitioner performance and patient outcomes: a synthesis of high-quality systematic review findings. Journal of the American Medical Informatics Association : JAMIA. 2011;18:327-34.

[12] Dutch Cochrane Centre. <http://www.cochrane.nl/Files/documents/Checklists/SR-RCT.pdf;> 2009

[13] Kang D, Wu Y, Hu D, Hong Q, Wang J, Zhang X. Reliability and External Validity of AMSTAR in Assessing Quality of TCM Systematic Reviews. Evidence-based complementary and alternative medicine : eCAM. 2012;2012:732195.

[14] P. Alderson SG, and J. P. T. Higgins, . Cochrane Reviewers’ Handbook 4.2.2, The Cochrane Library. Chichester, UK: John Wiley & Sons; 2004.

[15] Kitsiou S, Pare G, Jaana M. Effects of home telemonitoring interventions on patients with chronic heart failure: an overview of systematic reviews. Journal of medical Internet research. 2015;17:e63.

[16] Mahtani KR, Protheroe J, Slight SP, Demarzo MM, Blakeman T, Barton CA, et al. Can the London 2012 Olympics 'inspire a generation' to do more physical or sporting activities? An overview of systematic reviews. BMJ open. 2013;3.

[17] Higgins JPT GSe. Cochrane handbook for systematic reviews of interventions. Cochrane Collab 2011; Version 5.1.0 (updated March 2011). <http://www.cochrane-handbook.org;> [accessed 30 August 2018].

[18] McCall MC, Ward A, Roberts NW, Heneghan C. Overview of systematic reviews: yoga as a therapeutic intervention for adults with acute and chronic health conditions. Evidence-based complementary and alternative medicine : eCAM. 2013;2013:945895.

[19] Oxman AD. Checklists for review articles. Bmj. 1994;309:648-51.

[20] Mickenautsch S, Yengopal V. Extent and quality of systematic review evidence related to minimum intervention in dentistry: essential oils, powered toothbrushes, triclosan, xylitol. International dental journal. 2011;61:179-92.

[21] Moher D, Cook DJ, Eastwood S, Olkin I, Rennie D, Stroup DF. Improving the quality of reports of meta-analyses of randomised controlled trials: the QUOROM statement. Quality of Reporting of Meta-analyses. Lancet. 1999;354:1896-900.

[22] Pollock A, Farmer SE, Brady MC, Langhorne P, Mead GE, Mehrholz J, et al. Interventions for improving upper limb function after stroke. The Cochrane database of systematic reviews. 2014:CD010820.

[23] Popovich I, Windsor B, Jordan V, Showell M, Shea B, Farquhar CM. Methodological quality of systematic reviews in subfertility: a comparison of two different approaches. PloS one. 2012;7:e50403.

[24] Moher D, Liberati A, Tetzlaff J, Altman DG, Group P. Preferred reporting items for systematic reviews and meta-analyses: the PRISMA statement. Journal of clinical epidemiology. 2009;62:1006-12.

[25] Remes O, Brayne C, van der Linde R, Lafortune L. A systematic review of reviews on the prevalence of anxiety disorders in adult populations. Brain and behavior. 2016;6:e00497.

[26] Moher D, Liberati A, Tetzlaff J, Altman DG, Group P. Preferred reporting items for systematic reviews and meta-analyses: the PRISMA Statement. Open medicine : a peer-reviewed, independent, open-access journal. 2009;3:e123-30.

[27] Ryan R, Santesso N, Lowe D, Hill S, Grimshaw J, Prictor M, et al. Interventions to improve safe and effective medicines use by consumers: an overview of systematic reviews. The Cochrane database of systematic reviews. 2014:CD007768.

[28] Saez-Benito L, Fernandez-Llimos F, Feletto E, Gastelurrutia MA, Martinez-Martinez F, Benrimoj SI. Evidence of the clinical effectiveness of cognitive pharmaceutical services for aged patients. Age and ageing. 2013;42:442-9.

[29] Moher D, Liberati A, Tetzlaff J, Altman DG, Group P. Preferred reporting items for systematic reviews and meta-analyses: the PRISMA statement. Bmj. 2009;339:b2535.

[30] Tafelski S, Hauser W, Schafer M. Efficacy, tolerability, and safety of cannabinoids for chemotherapy-induced nausea and vomiting--a systematic review of systematic reviews. Schmerz. 2016;30:14-24.

[31] Hutton B, Salanti G, Caldwell DM, Chaimani A, Schmid CH, Cameron C, et al. The PRISMA extension statement for reporting of systematic reviews incorporating network meta-analyses of health care interventions: checklist and explanations. Annals of internal medicine. 2015;162:777-84.

[32] Tricco AC, Antony J, Vafaei A, Khan PA, Harrington A, Cogo E, et al. Seeking effective interventions to treat complex wounds: an overview of systematic reviews. BMC medicine. 2015;13:89.

[33] Windsor B, Popovich I, Jordan V, Showell M, Shea B, Farquhar C. Methodological quality of systematic reviews in subfertility: a comparison of Cochrane and non-Cochrane systematic reviews in assisted reproductive technologies. Human reproduction. 2012;27:3460-6.

[34] Egger M, Smith GD, Altman DG. Systematic Reviews in Health Care. London, UK: BMJ Publishing Group; 2001.

[35] Bidonde J, Busch AJ, Bath B, Milosavljevic S. Exercise for adults with fibromyalgia: an umbrella systematic review with synthesis of best evidence. Current rheumatology reviews. 2014;10:45-79.

[36] Moher D, Tetzlaff J, Tricco AC, Sampson M, Altman DG. Epidemiology and reporting characteristics of systematic reviews. PLoS medicine. 2007;4:e78.

[37] Chen Y, Sun J, Yang Y, Huang Y, Liu G. Malignancy risk of anti-tumor necrosis factor alpha blockers: an overview of systematic reviews and meta-analyses. Clinical rheumatology. 2016;35:1-18.

[38] Howells L, Musaddaq B, McKay AJ, Majeed A. Clinical impact of lifestyle interventions for the prevention of diabetes: an overview of systematic reviews. BMJ open. 2016;6:e013806.

[39] Wilczynski NL, Haynes RB, Hedges T. EMBASE search strategies achieved high sensitivity and specificity for retrieving methodologically sound systematic reviews. Journal of clinical epidemiology. 2007;60:29-33.

[40] Montori VM, Wilczynski NL, Morgan D, Haynes RB, Hedges T. Optimal search strategies for retrieving systematic reviews from Medline: analytical survey. Bmj. 2005;330:68.

[41] Kumar S, Beaton K, Hughes T. The effectiveness of massage therapy for the treatment of nonspecific low back pain: a systematic review of systematic reviews. International journal of general medicine. 2013;6:733-41.

[42] Liberati A, Altman DG, Tetzlaff J, Mulrow C, Gotzsche PC, Ioannidis JP, et al. The PRISMA statement for reporting systematic reviews and meta-analyses of studies that evaluate healthcare interventions: explanation and elaboration. Bmj. 2009;339:b2700.

[43] Macaya Pascual A, Ferreres Riera JR, Campoy Sanchez A. Behavioral Interventions for Preventing Sexually Transmitted Infections and Unintended Pregnancies: An Overview of Systematic Reviews. Actas dermo-sifiliograficas. 2016;107:301-17.

[44] Rotta I, Salgado TM, Silva ML, Correr CJ, Fernandez-Llimos F. Effectiveness of clinical pharmacy services: an overview of systematic reviews (2000-2010). International journal of clinical pharmacy. 2015;37:687-97.

[45] Moher D, Liberati A, Tetzlaff J, Altman DG, Group P. Preferred reporting items for systematic reviews and meta-analyses: the PRISMA statement. International journal of surgery. 2010;8:336-41.

[46] Wu X, Chung VC, Hui EP, Ziea ET, Ng BF, Ho RS, et al. Effectiveness of acupuncture and related therapies for palliative care of cancer: overview of systematic reviews. Scientific reports. 2015;5:16776.

[47] Korzeniewski SJ, Birbeck G, DeLano MC, Potchen MJ, Paneth N. A systematic review of neuroimaging for cerebral palsy. Journal of child neurology. 2008;23:216-27.

[48] National Health and Medical Research Council. How to Review the Evidence: Systematic Identification and Review of the Scientific Literature. Canberra, Australia: National Health and Medical Research Council; 1999.

[49] National Health and Medical Research Council. How to review the evidence: systematic identification and review of the scientific literature. Canberra: National Health and Medical Research Council; 2000.

[50] Willis BH, Quigley M. The assessment of the quality of reporting of meta-analyses in diagnostic research: a systematic review. BMC medical research methodology. 2011;11:163.

[51] Chalmers I, Altman DG. Systematic reviews. London: BMJ Publishing Group; 1995.

[52] Kastner M, Tricco AC, Soobiah C, Lillie E, Perrier L, Horsley T, et al. What is the most appropriate knowledge synthesis method to conduct a review? Protocol for a scoping review. BMC medical research methodology. 2012;12:114.

[53] Evans DK, Popova A, World B, Africa Regional O, Office of the Chief E. What really works to improve learning in developing countries? : an analysis of divergent findings in systematic reviews. [Washington, D.C.]: World Bank; 2015.

[54] The Campbell Collaboration. “What Is a Systematic Review? <http://www.campbellcollaboration.org/what_is_a_systematic_review/index.php;> 2015.

[55] Cochrane. What Is Cochrane Evidence and How Can It Help You? <http://www.cochrane.org/what-is-cochrane-evidence;> 2015.

[56] Haase SC. Systematic reviews and meta-analysis. Plastic and reconstructive surgery. 2011;127:955-66.

[57] Foisy M, Boyle RJ, Chalmers JR, Simpson EL, Williams HC. Overview of Reviews The prevention of eczema in infants and children: an overview of Cochrane and non-Cochrane reviews. Evidence-based child health : a Cochrane review journal. 2011;6:1322-39.

[58] Waters E, Doyle J, Jackson N, Howes F, Brunton G, Oakley A, et al. Evaluating the effectiveness of public health interventions: the role and activities of the Cochrane Collaboration. Journal of epidemiology and community health. 2006;60:285-9.

[59] Kapadia MZ, Askie L, Hartling L, Contopoulos-Ioannidis D, Bhutta ZA, Soll R, et al. PRISMA-Children (C) and PRISMA-Protocol for Children (P-C) Extensions: a study protocol for the development of guidelines for the conduct and reporting of systematic reviews and meta-analyses of newborn and child health research. BMJ open. 2016;6:e010270.

[60] Moher D, Shamseer L, Clarke M, Ghersi D, Liberati A, Petticrew M, et al. Preferred reporting items for systematic review and meta-analysis protocols (PRISMA-P) 2015 statement. Systematic reviews. 2015;4:1.

[61] Petrosino A, Lavenberg J, Mosteller F, Weisburd D, Finckenauer J, Clear T, et al. Systematic reviews and meta-analyses: Best evidence on “what works” for criminal justice decision makers2007.

[62] Khan KS, University of Y, Reviews NHSCf, Dissemination. Undertaking systematic reviews of research on effectiveness : CRD's guidelines for those carrying out or commissioning reviews. York: University of York, NHS Centre for Reviews and Dissemination; 2001.

[63] Robertson J, Hatton C, Baines S, Emerson E. Systematic Reviews of the Health or Health care of People with Intellectual Disabilities: A Systematic Review to Identify Gaps in the Evidence Base. Journal of applied research in intellectual disabilities : JARID. 2015;28:455-523.

[64] Shea BJ, Grimshaw JM, Wells GA, Boers M, Andersson N, Hamel C, et al. Development of AMSTAR: a measurement tool to assess the methodological quality of systematic reviews. BMC medical research methodology. 2007;7:10.

[65] Kaltenthaler E TPPS. NICE DSU Technical Support Document 13 Identifying and Reviewing Evidence to Inform the Conceptualisation and Population of Cost-Effectiveness Models. [S.l.]: National Institute for Health and Care Excellence (NICE); 2011.

[66] Lee JG, Ylioja T, Lackey M. Identifying Lesbian, Gay, Bisexual, and Transgender Search Terminology: A Systematic Review of Health Systematic Reviews. PloS one. 2016;11:e0156210.

[67] Bhurke S, Cook A, Tallant A, Young A, Williams E, Raftery J. Using systematic reviews to inform NIHR HTA trial planning and design: a retrospective cohort. BMC medical research methodology. 2015;15:108.

[68] Tsakalerou M. Systematic Reviews and Metastudies:

A Meta-Analysis Framework. International Journal of Science and Advanced Technology. 2015;5.

[69] Hansen H, Trifkovic N, Danmark, Danida, Danmark, Danida, et al. Systematic reviews questions, methods and usage. Copenhagen: Danida; 2013.

[70] Evidence-informed policy and practice (EPPI) centre: what is a systematic review?; 2009.

[71] The Campbell Collaboration. What Is a Systematic Review? <http://www.campbellcollaboration.org/what_is_a_systematic_review/index.php;> 2015.

[72] Cochrane Collaboration. [www.cochrane.org](http://www.cochrane.org).

[73] Weed DL. The quality of nutrition and cancer reviews: a systematic assessment. Critical reviews in food science and nutrition. 2013;53:276-86.

[74] Winter DA, British Association for C, Psychotherapy. Counselling and psychotherapy for the prevention of suicide : a systematic review of the evidence. Lutterworth [England]: BACP; 2009.

[75] University of Y, Reviews NHSCf, Dissemination. Undertaking systematic reviews of research on effectiveness : CRD's guidance for those carrying out or commissioning reviews. York, England: Centre for Reviews and Dissemination, University of York; 2001.

[76] Soares CB, Hoga LA, Peduzzi M, Sangaleti C, Yonekura T, Silva DR. [Integrative review: concepts and methods used in nursing]. Revista da Escola de Enfermagem da U S P. 2014;48:335-45.

[77] van der Linde RM, Stephan BC, Savva GM, Dening T, Brayne C. Systematic reviews on behavioural and psychological symptoms in the older or demented population. Alzheimer's research & therapy. 2012;4:28.

[78] Aveyard H. Doing a literature review in health and social care : a practical guide. Maidenhead: Open University Press; 2014.

[79] Mulrow CD, Cook DJ, Davidoff F. Systematic Reviews: Critical Links in the Great Chain of Evidence. Annals of internal medicine. 1997;126:389-90.

[80] Ilomaki J, Jokanovic N, Tan EC, Lonnroos E. Alcohol Consumption, Dementia and Cognitive Decline: An Overview of Systematic Reviews. Current clinical pharmacology. 2015;10:204-12.

[81] Oestergaard S, Moldrup C. Improving outcomes for patients with depression by enhancing antidepressant therapy with non-pharmacological interventions: a systematic review of reviews. Public health. 2011;125:357-67.

[82] Akram Y, Copello A, Moore D. Family-based interventions for substance misuse: a systematic review of systematic reviews--protocol. Systematic reviews. 2014;3:90.

[83] Borge CR, Hagen KB, Mengshoel AM, Omenaas E, Moum T, Wahl AK. Effects of controlled breathing exercises and respiratory muscle training in people with chronic obstructive pulmonary disease: results from evaluating the quality of evidence in systematic reviews. BMC pulmonary medicine. 2014;14:184.

[84] Shea BJ, Hamel C, Wells GA, Bouter LM, Kristjansson E, Grimshaw J, et al. AMSTAR is a reliable and valid measurement tool to assess the methodological quality of systematic reviews. Journal of clinical epidemiology. 2009;62:1013-20.

[85] Rogante M, Kairy D, Giacomozzi C, Grigioni M. A quality assessment of systematic reviews on telerehabilitation: what does the evidence tell us? Annali dell'Istituto superiore di sanita. 2015;51:11-8.

[86] Shikora SA, Mahoney CB. Clinical Benefit of Gastric Staple Line Reinforcement (SLR) in Gastrointestinal Surgery: a Meta-analysis. Obesity surgery. 2015;25:1133-41.

[87] Thomas MJW, Australian Transport Safety B, Westwood-Thomas A. A systematic review of the effectiveness of safety management systems. Canberra, A.C.T.: Australian Transport Safety Bureau; 2012.

[88] Faggion CM, Jr., Listl S, Giannakopoulos NN. The methodological quality of systematic reviews of animal studies in dentistry. Veterinary journal. 2012;192:140-7.

[89] Vigna-Taglianti F, Vineis P, Liberati A, Faggiano F. Quality of systematic reviews used in guidelines for oncology practice. Annals of oncology : official journal of the European Society for Medical Oncology. 2006;17:691-701.

[90] Fleming PS, Seehra J, Polychronopoulou A, Fedorowicz Z, Pandis N. Cochrane and non-Cochrane systematic reviews in leading orthodontic journals: a quality paradigm? European journal of orthodontics. 2013;35:244-8.

[91] Sequeira-Byron P, Fedorowicz Z, Jagannath VA, Sharif MO. An AMSTAR assessment of the methodological quality of systematic reviews of oral healthcare interventions published in the Journal of Applied Oral Science (JAOS). Journal of applied oral science : revista FOB. 2011;19:440-7.

[92] Sander L, Kitcher H, National Institute for H, Clinical E. Systematic and other reviews : terms and definitions used by UK organisations and selected database ; systematic review and Delphi survey. London: National Institute for Health and Clinical Excellence; 2006.

[93] Hunter C, Januszyk M, Wan DC, Momeni A. Systematic Reviews in Craniofacial Trauma-Strengths and Weaknesses. Annals of plastic surgery. 2016;77:363-8.

[94] Momeni A, Jacobson JY, Lee GK. Systematic reviews addressing microsurgical head and neck reconstruction. The Journal of craniofacial surgery. 2015;26:210-3.

[95] Momeni A, Lee GK, Talley JR. The quality of systematic reviews in hand surgery: an analysis using AMSTAR. Plastic and reconstructive surgery. 2013;131:831-7.

[96] Lundh A, Knijnenburg SL, Jorgensen AW, van Dalen EC, Kremer LC. Quality of systematic reviews in pediatric oncology--a systematic review. Cancer treatment reviews. 2009;35:645-52.

[97] Dutch Cochrane Centre. <http://www.cochrane.nl/Files/documents/Checklists/SR-RCT.pdf;> 2009.

[98] Alderson P, Green S, Higgins JPT. Cochrane Reviewers’ Handbook 4.2.2, The Cochrane Library, Issue 1, John Wiley & Sons, Chichester, UK, 2004.

[99] Laver K, Dyer S, Whitehead C, Clemson L, Crotty M. Interventions to delay functional decline in people with dementia: a systematic review of systematic reviews. BMJ open. 2016;6:e010767.

[100] Martinez-Gonzalez NA, Berchtold P, Ullman K, Busato A, Egger M. Integrated care programmes for adults with chronic conditions: a meta-review. International journal for quality in health care : journal of the International Society for Quality in Health Care. 2014;26:561-70.

[101] O'Connell NE, Wand BM, McAuley J, Marston L, Moseley GL. Interventions for treating pain and disability in adults with complex regional pain syndrome. The Cochrane database of systematic reviews. 2013:CD009416.

[102] Plaszewski M, Bettany-Saltikov J. Non-surgical interventions for adolescents with idiopathic scoliosis: an overview of systematic reviews. PloS one. 2014;9:e110254.

[103] Plaszewski M, Bettany-Saltikov J. Are current scoliosis school screening recommendations evidence-based and up to date? A best evidence synthesis umbrella review. European spine journal : official publication of the European Spine Society, the European Spinal Deformity Society, and the European Section of the Cervical Spine Research Society. 2014;23:2572-85.

[104] The Cochrane Collaboration. Glossary of Terms in The Cochrane Collaboration. Version 4.2.5. Available: <http://www.cochrane.org/sites/default/files/uploads/glossary.pdf;> 2005.

[105] Sekhon M, Cartwright M, Francis JJ. Acceptability of healthcare interventions: an overview of reviews and development of a theoretical framework. BMC health services research. 2017;17:88.

[106] Savard LA, Thompson DR, Clark AM. A meta-review of evidence on heart failure disease management programs: the challenges of describing and synthesizing evidence on complex interventions. Trials. 2011;12:194.

[107] Whitlock EP, Lin JS, Chou R, Shekelle P, Robinson KA. Using existing systematic reviews in complex systematic reviews. Annals of internal medicine. 2008;148:776-82.

[108] Yucel E, Sancar M, Yucel A, Okuyan B. Adverse drug reactions due to drug-drug interactions with proton pump inhibitors: assessment of systematic reviews with AMSTAR method. Expert opinion on drug safety. 2016;15:223-36.

[109] Liberati A, Altman DG, Tetzlaff J, Mulrow C, Gotzsche PC, Ioannidis JP, et al. The PRISMA statement for reporting systematic reviews and meta-analyses of studies that evaluate health care interventions: explanation and elaboration. Annals of internal medicine. 2009;151:W65-94.

[110] Page MJ, McKenzie JE, Green SE, Forbes AB. An empirical investigation of the potential impact of selective inclusion of results in systematic reviews of interventions: study protocol. Systematic reviews. 2013;2:21.

[111] Beller EM, Glasziou PP, Hopewell S, Altman DG. Reporting of effect direction and size in abstracts of systematic reviews. Jama. 2011;306:1981-2.

[112] Beller EM, Chen JK, Wang UL, Glasziou PP. Are systematic reviews up-to-date at the time of publication? Systematic reviews. 2013;2:36.

[113] Faggion CM, Jr., Liu J, Huda F, Atieh M. Assessment of the quality of reporting in abstracts of systematic reviews with meta-analyses in periodontology and implant dentistry. Journal of periodontal research. 2014;49:137-42.

[114] Lu LY, Zheng GQ, Wang Y. An overview of systematic reviews of shenmai injection for healthcare. Evidence-based complementary and alternative medicine : eCAM. 2014;2014:840650.

[115] Abalos E, Carroli G, Mackey ME, Bergel E. Critical appraisal of systematic reviews: the WHO reproductive health library 4WHO/RHR/01.6, The World Health Organization, Geneva, Switzerland, 2001.

[116] Ernst E, Lee MS, Choi TY. Acupuncture for insomnia? An overview of systematic reviews. The European journal of general practice. 2011;17:116-23.

[117] Smith V, Devane D, Begley CM, Clarke M. Methodology in conducting a systematic review of systematic reviews of healthcare interventions. BMC medical research methodology. 2011;11:15.

[118] Bowling A, Dawsonera. Research methods in health investigating health and health services2014.

[119] Bambra C. Real world reviews: a beginner's guide to undertaking systematic reviews of public health policy interventions. Journal of epidemiology and community health. 2011;65:14-9.

[120] Oakley A, Fullerton D. The lamp-post of research: support or illumination? In: Oakley A, Roberts H, eds. Evaluating social interventions: a report of two workshops funded by the Economic and Social Research Council. Ilford: Barnardos, 1998:4e38.

[121] Jesson J, Lacey F. How to do (or not to do) a critical literature review. Pharmacy Education. 2006;6:139-48.

[122] Khan KS, Kunz R, Kleijnen J, Antes G. Systematic reviews to support evidence-based medicine. In: Petti (Ed.), Metaanalysis, decision analysis and cost effectiveness analysis. Methods in quantitative synthesis in medicine. Oxford: Oxford University Press; 2003.

[123] Mueller KF, Briel M, Strech D, Meerpohl JJ, Lang B, Motschall E, et al. Dissemination bias in systematic reviews of animal research: a systematic review. PloS one. 2014;9:e116016.

[124] Peters JL, Sutton AJ, Jones DR, Rushton L, Abrams KR. A systematic review of systematic reviews and meta-analyses of animal experiments with guidelines for reporting. Journal of environmental science and health Part B, Pesticides, food contaminants, and agricultural wastes. 2006;41:1245-58.

[125] Korevaar DA, Hooft L, ter Riet G. Systematic reviews and meta-analyses of preclinical studies: publication bias in laboratory animal experiments. Laboratory animals. 2011;45:225-30.

[126] Potting C, Mistiaen P, Poot E, Blijlevens N, Donnelly P, van Achterberg T. A review of quality assessment of the methodology used in guidelines and systematic reviews on oral mucositis. Journal of clinical nursing. 2009;18:3-12.

[127] The Cochrane Collaboration. The Cochrane Library: Evidence for Healthcare Decision-Making. Available at: <http://www3.interscience.wiley.com/cgi-bin/mrwhome/106568753/whataresystematicreviews.html.;> 2007.

[128] Iwarsson E, Jacobsson B, Dagerhamn J, Davidson T, Bernabe E, Heibert Arnlind M. Analysis of cell-free fetal DNA in maternal blood for detection of trisomy 21, 18 and 13 in a general pregnant population and in a high risk population - a systematic review and meta-analysis. Acta obstetricia et gynecologica Scandinavica. 2017;96:7-18.

[129] Cochrane Library. [www.thecochranelibrary.com](http://www.thecochranelibrary.com).

[130] Neto ACD, Subramanyan R, Vieira M, Travassos GH. Characterization of Model-based SoftwareTesting Approaches. Available at: https://[www.cos.ufrj.br/uploadfile/1188491168.pdf;](http://www.cos.ufrj.br/uploadfile/1188491168.pdf;) 2007.

[131] Baldassarre MT. A Hands-On Approach for Teaching Systematic Review. In: Jedlitschka A, Salo O. Product-Focused Software Process Improvement; 2008, p. 415–426.

[132] Kitchenham B. Procedures for Performing Systematic Review, Joint Technical Report Software Engineering Group, Department of Computer Science Keele University, United King and Empirical Software Engineering, National ICT Australia Ltd, Australia; 2004.

[133] Lichtner V, Dowding D, Esterhuizen P, Closs SJ, Long AF, Corbett A, et al. Pain assessment for people with dementia: a systematic review of systematic reviews of pain assessment tools. BMC geriatrics. 2014;14:138.

[134] Zwakhalen SM, Hamers JP, Abu-Saad HH, Berger MP. Pain in elderly people with severe dementia: a systematic review of behavioural pain assessment tools. BMC geriatrics. 2006;6:3.

[135] Liberati A, Altman DG, Tetzlaff J, Mulrow C, Gotzsche PC, Ioannidis JP, et al. The PRISMA statement for reporting systematic reviews and meta-analyses of studies that evaluate health care interventions: explanation and elaboration. PLoS medicine. 2009;6:e1000100.

[136] Pope C, Mays N, Popay J. How can we synthesize qualitative and quantitative evidence for healthcare policy-makers and managers? Healthcare management forum. 2006;19:27-31.

[137] The Jenner Institute. <http://www.jr2.ox.ac.uk/bandolier/booth/diagnos/glossary.html>.

[138] Daigneault P-M. Taking stock of four decades of quantitative research on stakeholder participation and evaluation use: A systematic map. Evaluation and Program Planning Evaluation and Program Planning. 2014;45:171-81.

[139] Petticrew M, Roberts H. Systematic reviews in the social sciences : a practical guide. 2006.

[140] Ranson K, Law TJ, Bennett S. Establishing health systems financing research priorities in developing countries using a participatory methodology. SSM Social Science & Medicine. 2010;70:1933-42.

[141] Lavis JN, Hammill AC, Wilson MG, Boyko JA, Grimshaw J, Oxman AD. Systematic reviews that can inform health systems management and policymaking ;2007.

[142] Marinho VCC. Systematic reviews of controlled trials in general and oral health care. Braz J Oral Sci. 2003 2.

[143] Mulrow CD, Cook DJ, Davidoff F. Systematic reviews: critical links in the great chain of evidence. Annals of internal medicine. 1997;126:389-91.

[144] University of Y, Reviews NHSCf, Dissemination. Undertaking systematic reviews of research on effectiveness. CRD Guidelines for those carrying out or commissioning reviews: University of York; 1996.

[145] Newcomer KE, Wholey JS, Hatry HP. Handbook of practical program evaluation, 4th ed. San Francisco, Calif.: Jossey-Bass; 2015.

[146] Chalmers I, Hedges LV, Cooper H. A brief history of research synthesis. Evaluation & the health professions. 2002;25:12-37.

[147] Moher D, Liberati A, Tetzlaff J, Altman DG, Group P. Preferred reporting items for systematic reviews and meta-analyses: the PRISMA statement. Annals of internal medicine. 2009;151:264-9, W64.

[148] Sanchez-Puerta ML, Valerio A, Bernal MG. Taking stock of programs to develop socioemotional skills : a systematic review of program evidence2016.

[149] Waddington H, White H, Snilstveit B, Hombrados JG, Vojtkova M, Davies P, et al. How to do a good systematic review of effects in international development: A tool kit. J Dev Eff Journal of Development Effectiveness. 2012;4:359-87.

[150] Baude W, Chilton AS, Malani A. Making Doctrinal Work More Rigorous: Lessons from Systematic Reviews. University of Chicago law review. 2017;84:37-58.

[151] Garg AX, Hackam D, Tonelli M. Systematic review and meta-analysis: When one study is just not enough. Clin J Am Soc Nephrol Clinical Journal of the American Society of Nephrology. 2008;3:253-60.

[152] Franco, MF. Physicians’ attitudes towards human papillomavirus vaccination programme: A systematic review. University of Chester; 2011.

[153] Best A, Greenhalgh T, Lewis S, Saul JE, Carroll S, Bitz J. Large-System Transformation in Health Care: A Realist Review <i>Large-System Transformation in Health Care</i>. Milbank Quarterly. 2012;90:421-56.

[154] Greenhalgh T, Robert G, Macfarlane F, Bate P, Kyriakidou O. Diffusion of Innovations in Service Organizations: Systematic Review and Recommendations. MILQ Milbank Quarterly. 2004;82:581-629.

[155] Maitland A, Hills LA, Rhind DJ. Organisational culture in sport - A systematic review. Sport management review. 2015;18:501-16.

[156] Klassen TP, Jadad AR, Moher D. Guides for Reading and Interpreting Systematic Reviews I. Getting Started. Arch Pediatr Adolesc Med Archives of Pediatrics & Adolescent Medicine. 1998;152.

[157] Svejvig P, Andersen P. Rethinking project management: A structured literature review with a critical look at the brave new world. JPMA International Journal of Project Management. 2015;33:278-90.

[158] Shukla M, Jharkharia S. Agri-fresh produce supply chain management: a state-of-the-art literature review. INTERNATIONAL JOURNAL OF OPERATIONS AND PRODUCTION MANAGEMENT. 2013;33:114-58.

[159] Bastos LdSL, Mendes ML, Nunes DRdL, Melo ACS, Carneiro MP. A systematic literature review on the joint replenishment problem solutions: 2006-2015. Prod Production. 2017;27.

[160] Tranfield D, Denyer D, Smart P. Towards a Methodology for Developing Evidence-Informed Management Knowledge by Means of Systematic Review. BRITISH JOURNAL OF MANAGEMENT. 2003;14:207-22.

[161] Neale J. Research methods for health and social care. New York, NY.: Palgrave MacMillan; 2009.

[162] Oxman AD, Guyatt GH. Guidelines for reading literature reviews. CMAJ : Canadian Medical Association journal = journal de l'Association medicale canadienne. 1988;138:697-703.

[163] Sjöholm J, Luleå tekniska u, Institutionen för samhällsbyggnad och n. Built cultural heritage in an urban planning context Literature review. Lulea: Luleå tekniska universitet; 2013.

[164] Sjöholm J. Heritagisation of built environments : a study of the urban transformation in Kiruna, Sweden. Luleå: Division of Architecture and Water, Luleå University of Technology; 2013.

[165] Jesson J, Matheson L, Lacey FM. Doing your literature review : traditional and systematic techniques. Los Angeles: SAGE; 2011.

[166] Matney, WB. The use of percussion in therapy: a realist synthesis. The Dissertation Committee for Bill Matney certifies; 2015.

[167] Gough D, Oliver S, Thomas J. An introduction to systematic reviews. London; Thousand Oaks, Calif.: Sage; 2012.

[168] Hanson-Abromeit D, Sena Moore K. The systematic review as a research process in music therapy. Journal of music therapy. 2014;51:4-38.

[169] Porta MS, Greenland S, Last JM. A dictionary of epidemiology. New York; Oxford: Oxford University Press; 2008.

[170] Khan K, Kunz R, Kleijnen J, Khan KS. Systematic Reviews to Support Evidence-Based Medicine. London, GBR: CRC Press; 2011.

[171] Marshall, C. Tool support for systematic reviews in software engineering; 2016.

[172] Marshall C. Tool Features to Support Systematic Reviews in Software Engineering – A Cross Domain Study. e-Informatica Software Engineering Journal. 2018;12:79-115.

[173] Mulrow CD. Rationale for systematic reviews. British medical journal. 1994;309:597.

[174] Cook DJ, Mulrow CD, Haynes RB. Systematic Reviews: Synthesis of Best Evidence for Clinical Decisions. Annals of internal medicine. 1997;126:376-80.

[175] Ba K, Charters S. Guidelines for performing Systematic Literature Reviews in Software Engineering2007.

[176] Haddaway NR, Pullin AS. The Policy Role of Systematic Reviews: Past, Present and Future. Springer Science Reviews Springer Science Reviews. 2014;2:179-83.

[177] Thomé AMT, Scavarda LF, Scavarda AJ. Conducting systematic literature review in operations management. Production Planning & Control Production Planning & Control. 2016;27:408-20.

[178] Hall HR, Roussel L. Evidence-based practice : an integrative approach to research, administration, and practice. 2017.

[179] Littell JH, Corcoran J, Pillai VK. Systematic reviews and meta-analysis. Oxford; New York: Oxford University Press; 2008.

[180] Higgins JPT, Green S. Cochrane handbook of systematic reviews of interventions. Chichester: Wiley; 2008.

[181] Antman EM. A comparison of results of meta-analyses of randomized control trials and recommendations of clinical experts. Treatments for myocardial infarction. JAMA: The Journal of the American Medical Association. 1992;268:240-8.

[182] Oxman AD, Guyatt GH. The science of reviewing research. Annals of the New York Academy of Sciences. 1993;703:125-33.

[183] Holly C, Salmond SW, Saimbert M. Comprehensive systematic review for advanced nursing practice. New York: Springer Pub.; 2012.

[184] Centre for R, Dissemination. Systematic reviews CRD's guidance for undertaking reviews in health care. York: CRD; 2009.

[185] O'Rourke, A. Critical appraisal. In: Bowling, A, Ebrahim, S. Handbook of Health Research Methods: Investigation, Measurement and Analysis. Maiden head: Open university press; 2005.

[186] Sharma R, Gordon M, Dharamsi S, Gibbs TJ, Association for Medical Education in E. Systematic reviews in medical education : a practical approach2015.

[187] Crowther MA, Cook DJ. Trials and tribulations of systematic reviews and meta-analyses. Hematology American Society of Hematology Education Program. 2007:493-7.

[188] Khan KS KR, Kleijnen J, Antes G. Five steps to conducting a systematic review. J R Soc Med Journal of the Royal Society of Medicine. 2003;96:118-21.

[189] Evidence-Based Behavioral Practice. Systematic review module: Introduction to systematic reviews. <http://www.ebbp.org/training.html;> 2013.
