# Supplementary material for: Definition of a systematic review used in overviews of systematic reviews, meta-epidemiological studies and textbooks
Source: BMC Med Res Methodol. 2019 Nov 4;19:203. doi: 10.1186/s12874-019-0855-0 (PMC6829801; doi:10.1186/s12874-019-0855-0)
Supplement: Supplementary file 2 — Additional file 2: Table S3. Definitions of a systematic review from nine manuscripts with the most commonly used combination of categories. A file containing verbatim extracted definitions that were used in nine manuscripts that had the most commonly used combination of categories of systematic review definitions [file 12874_2019_855_MOESM2_ESM.docx]

**Supplementary table 3. Definitions of a systematic review from nine manuscripts with the most commonly used combination of categories**

**Manuscript:** Howard-Wilsher, S., et al., Systematic overview of economic evaluations of health-related rehabilitation. Disabil Health J, 2016. 9(1): p. 11-25.

**Definition**: "To be included, an SR should have:

Defined review question(s) and inclusion/exclusion criteria that are relevant in terms of the PICO framework (cost-effectiveness or cost-benefits of health-related rehabilitation)

Reported the literature search strategy

Assessed study quality or risk of bias in results of the included economic evaluation studies

Provided the main characteristics of the included studies"

**Reference**: none

**Manuscript:** Long, L., et al., What is the clinical effectiveness and cost-effectiveness of conservative interventions for tendinopathy? An overview of systematic reviews of clinical effectiveness and systematic review of economic evaluations. Health Technol Assess, 2015. 19(8): p. 1-134.

**Definition**: For the purpose of this review, a systematic review was defined as one that has a focused research question; explicit search criteria that are available to review, either in the document or on application; explicit inclusion/exclusion criteria; definitions of the population(s), intervention(s), comparator(s) and outcome(s) of interest; a critical appraisal of included studies, including consideration of internal and external validity of the research; and a synthesis of the included evidence, whether narrative or quantitative.

**Reference**: none

**Manuscript:** John Lavis et al. Towards systematic reviews that inform health care management and policy-making. J Health Serv Res Policy Vol 10 Suppl 1 July 2005 S1:35.

**Definition**: By systematic reviews, we mean reviews of the research literature with five components: an explicit question; an explicit description of the search strategy; an explicit statement about what types of research evidence were included and excluded; a critical examination of the quality of the studies included in the review; and a critical and transparent process of interpretation of the findings of the studies included in the review.(…) "

**Reference**: none

**Manuscript:** Scott Shikora et al. Clinical Benefit of Gastric Staple Line Reinforcement (SLR) in Gastrointestinal Surgery: a Meta-analysis. OBES SURG (2015) 25:1133-1141. **Definition**: This study conforms to the standard definition of a systematic review, „A systematic review collates all empirical evidence regarding a particular research question, the key characteristics being:

-a clearly stated set of objectives (pre-defined eligibility criteria for studies);

-an explicit, reproducible methodology;

-a systematic search that attempts to identify all studies that would meet the eligibility criteria;

-an assessment of the validity of the findings of the included studies, e.g. assessment of risk of bias;

-a systematic presentation, and synthesis, of the characteristics and findings of the included studies. Many systematic reviews contain meta-analyses.“

**Reference**: Cochrane Handbook

**Manuscript:** Pierre Pluye et al. Opening-up the definition of systematic literature review: the plurality of worldviews, methodologies and methods for reviews and syntheses (Journal of Clinical Epidemiology 73, 2016, 2-5.

**Definition**: This article suggests an issue for which consensus is needed: the definition of a systematic review. The term ‘‘systematic’’ is broadly used, but rarely defined: some researchers seem to reserve this term for systematic reviews of randomized controlled trials; others seem to use this term as a talisman for publication and recognition and report nonsystematic reviews of convenience samples of studies (cherry-picking few articles in one bibliographic database and personal files without clear criteria). In line with the plurality of evidence used in health systems, we propose the following three-part definition. **A systematic review (1) is** explicit and transparent; (2) includes a type of research (empirical, methodological, theoretical) and a type of study method (qualitative, quantitative, mixed) or a combination of types; and (3) consists of a reproducible process with specific question(s) (exploratory or confirmatory or both), precise eligibility criteria, a comprehensive set of sources of information, an exhaustive search strategy (designed with specialized librarians), a reliable or dependable selection of relevant articles (based on relevance criteria that may differ between quantitative [all studies] and qualitative reviews [purposeful sample]), a quality appraisal of included studies and data extraction (using appropriate validated criteria for each type of design of included studies), which are performed by at least two researchers, and a rigorous synthesis (using qualitative or quantitative or mixed synthesis methods). The systematic review process may be iterative in qualitative reviews and reviews including qualitative, quantitative, and mixed-methods studies.

**Reference**: none

**Manuscript**: Lichtner et al. Pain assessment for people with dementia: a systematic review of systematic reviews of pain assessment tools. BMC Geriatrics 2014, 14:138.

**Definition** of systematic review:

1. Review carried out systematically –i.e. publication that makes explicit the authors’ intention to review or summarise the literature (e.g. with review, overview, or meta-analysis in the title or in a section heading) [21].

2. Satisfying the following criteria [22]:

- Clear set of objectives: explicit and clear research question

- Reproducible methodology: the paper clearly explains how the evidence was

retrieved, including sources and search strategy and the inclusion (and exclusion) criteria

- Assessment of validity of the findings (e.g. assessment of risk of bias)

- Systematic presentation and synthesis of findings beyond those provided by single studies.

**References**:

21.Zwakhalen S, Hamers J, Abu-Saad H, Berger M: Pain in elderly people with severe dementia: a systematic review of behavioural pain assessment

tools. BMC Geriatr 2006, 6(1):3.

22. Liberati A, Altman DG, Tetzlaff J, Mulrow C, Gotzsche PC, Ioannidis JP, Clarke M,

Devereaux PJ, Kleijnen J, Moher D: The PRISMA statement for reporting

systematic reviews and meta-analyses of studies that evaluate health care interventions: explanation and elaboration. PLoS medicine 2009, 6(7):e1000100.

**Manuscript**: M Hoyle et al. The clinical effectiveness and cost-effectiveness of cetuximab (mono- or combination chemotherapy), bevacizumab (combination with non-oxaliplatin chemotherapy) and panitumumab (monotherapy) for the treatment of metastatic colorectal cancer after first-line chemotherapy (review of technology appraisal No.150 and part review of technology appraisal No. 118): a systematic review and economic model. Health Technology Assessment, 2013; 17(14).

**Definition**: "For the purpose of this review, a systematic review was defined as one that has:

-a focused research question

-explicit search criteria that are available to review, either in the document or on application

-explicit inclusion/exclusion criteria, defining the population(s), intervention(s),

comparator(s) and outcome(s) of interest

-a critical appraisal of included studies, including consideration of the internal and external

validity of the research

-a synthesis of the included evidence, whether narrative or quantitative."

**Reference**: none

**Manuscript**: Tristan Snowsill et al. A systematic review and economic evaluation of diagnostic strategies for Lynch syndrome, HEALTH TECHNOLOGY ASSESSMENT 2014

VOL. 18 NO. 58.

**Definition**. "Systematic reviews were used as a source for finding further studies and to compare with our systematic review. For the purpose of this review, a systematic review was defined as one that has:

-a focused research question

-explicit search criteria that are available to review, either in the document or on application

-explicit inclusion/exclusion criteria, defining the population(s), intervention(s), comparator(s) and outcome(s) of interest

-a critical appraisal of included studies, including consideration of internal and external validity of the research

-a synthesis of the included evidence, whether narrative or quantitative."

**Reference**: none

**Book**: Athanasiou-Evidence synthesis in healthcare

**Definition**: A systematic review is defined as the objective, transparent and unbiased location and critical appraisal of the complete scope of research in a given topic and the eventual impartial synthesis and, if possible, meta-analysis of individual study findings. Therefore, in order to address, a specific research aim, a systematic review collates all evidence that fits pre-specified eligibility criteria.

The aim of a systematic review are manifold and includes the following:

Critical appraisal of individual studies

Combination of individual results to create an useful summary statistics

Analysis for presence of and reason behind between-study variances

Exposure of areas of research which might be methodologically inadequate and require further refinement

Exposure of knowledge gaps and areas of potential future research possibilities

Every systematic review is composed of a discrete number of steps:

formulation of a specific question to be addressed with a clearly stated set of objectives

Definition of eligibility (inclusion end exclusion) criteria for primary studies to be included Systematic search which identifies and locates all potentially eligible relevant studies whether published or unpublished

Performance of a variety of statistical methods to assess for heterogenity between studies

Impartial unbiased analysis and assessment of the validity of the results

Creation of a structured presentation, and synthesis to state and discuss upon findings and characteristics of collected information

**Reference**: none
